# Chemo- and regio-selective enzymatic lipophilisation of rutin, and physicochemical and antioxidant properties of rutin ester derivatives†

**DOI:** 10.1039/d3ra06333j

**Published:** 2023-12-04

**Authors:** Nikitia Mexia, Meryem Benohoud, Christopher M. Rayner, Richard S. Blackburn

**Affiliations:** a Leeds Institute of Textiles and Colour, School of Design, University of Leeds Leeds LS2 9JT UK r.s.blackburn@leeds.ac.uk; b School of Chemistry, University of Leeds Leeds LS2 9JT UK; c Keracol Limited Nexus, Discovery Way Leeds LS2 3AA UK

## Abstract

Enzymes are one of the most powerful tools in organic Green Chemistry and enzymatic reactions offer numerous advantages like regio- and enantio-selectivity along with their eco-friendly and sustainable nature. More specifically, lipases can catalyse both ester hydrolysis and formation depending on the nature of the substrate and water content. Herein, the focus is on the development of an enzymatically catalysed lipophilisation of natural compounds using lipases of microbial origin and the investigation of the optimal reaction conditions, aiming ultimately to ameliorate the compounds' properties. The flavonoid disaccharide rutin (quercetin-3-*O*-rutinoside) was the model compound on which the acylation protocol was built, allowing an efficient procedure to be established, while simultaneously offering the possibility of developing rapid, clear and robust methodologies, using state-of-the-art techniques, for analysis and purification of the synthesized compounds. An optimal 72 h reaction at 55 °C, using *Candida antarctica* lipase B immobilized on acrylic resin, combined with silicon dioxide as dehydrating agent, followed by product purification, achieved conversion ratios up to 50%. Full characterization and evaluation of the physicochemical and antioxidant properties of the esterified compounds was obtained. The lipophilicity of the rutin esters produced increased with increasing alkyl chain length, yet antioxidant properties were unaffected in comparison with the parent compound. A preparatively useful acylation protocol was established, allowing full investigation into the properties of the acylated compounds. It is also applicable for use on mixtures of compounds as most natural products are found in nature in mixtures and such a development greatly enhances the potential of this method for future commercial applications.

## Abstract 2

Enzymatic acylation of the flavonoid disaccharide rutin was achieved using *Candida antarctica* lipase B. Lipophilicity of the rutin esters produced increased with increasing alkyl chain length, yet antioxidant properties were unaffected.

## Graphical abstract

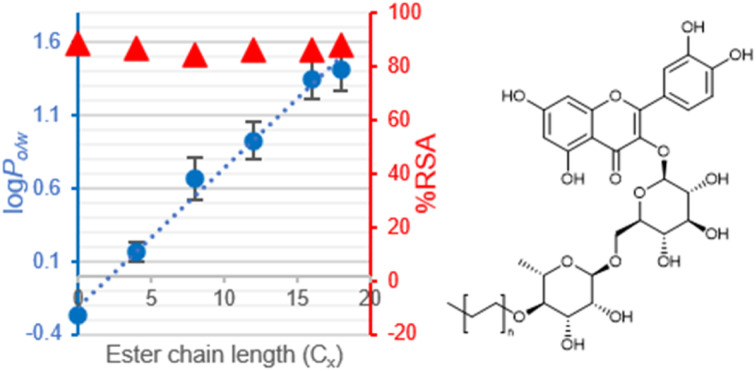

## 1. Introduction

Plant secondary metabolites are in most cases responsible for their organoleptic characteristics (*e.g.* odour, colour) and for the bioactivity of their extracts; one of the main families of compounds distributed in medicinal plants are flavonoids, with five major scaffolds in this family: chalcones, flavanones, flavones, flavonols and anthocyanins.^1^ Flavonoids are known for their pharmacological properties, including antioxidant activity while many also have potential to act as colorants.^2,3^ However, most flavonoids suffer from a lack of solubility in non-polar media (*e.g.* oils) and relatively low stability to light and oxidation, which limits their application in food, cosmetics and pharmaceuticals.^1,4,5^

Several methods are described in the literature for enhancing the stability and/or lipophilicity of these compounds, and one common method is acylation of free hydroxyls of the sugar moieties in the flavonoid glycosides.^4,6^ This reaction is considered to be the last step in the biosynthesis of flavonoids and it has been demonstrated, particularly in the case of anthocyanins, that acylated derivatives are more stable than their non-esterified counterparts, with differences noted between aliphatic and aromatic ester groups; aliphatic groups generally decrease flavonoid solubility in aqueous media, whereas aromatic acyl groups offer greater stability due to intramolecular co-pigmented complexation.^6,7^ Chemical acylation of glycosidic natural products can be non-selective,^8^ and the lack of control leads to reactions on multiple hydroxyl groups resulting in multi-acylation and formation of mono-, di, and tri-ester derivatives that can prove difficult to separate,^9^ with potentially decreased biological activity.^10^

In contrast, regioselective synthesis of flavonoid ester derivatives has been achieved by enzymatic catalysis;^11^ lipases of various origins have been used extensively for ester formation and hydrolysis, with high levels of selectivity, including enantioselectivity,^12^ depending upon the nature of the substrate and water content.^11^ Enzymatic acylations of isoquercetin (quercetin-3-*O*-glucoside; 1)^3,8,13,14^ and anthocyanin glucosides^1,6,9,15,16^ have been shown to be more chemo- and regioselective, and to enhance flavonoid solubility in less polar media, their stability,^17^ and biological activity.^18–20^ Successful enzymatic esterification using *Candida antarctica* lipase B (*Ca*lB) has been reported where the site of acylation is the glucose primary hydroxyl group. Reaction times reported in the literature for these enzymatic acylations are typically longer than 24 h, but application of microwave irradiation was found to reduce time and energy in regioselective enzymatic acylation of isoquercetin with long chain saturated (C_18_), mono- (C_18:1_), and poly-unsaturated (C_18:2_, C_18:3_, C_20:5_, C_22:6_) fatty acids; 85–98% yields were obtained in 2–3 min.^13^ Sonication-assisted lipase catalysed acylation of isoquercetin was also found to be more efficient and economical than conventional reaction conditions, reducing time required by 80% and giving yields of 81–96%.^14^
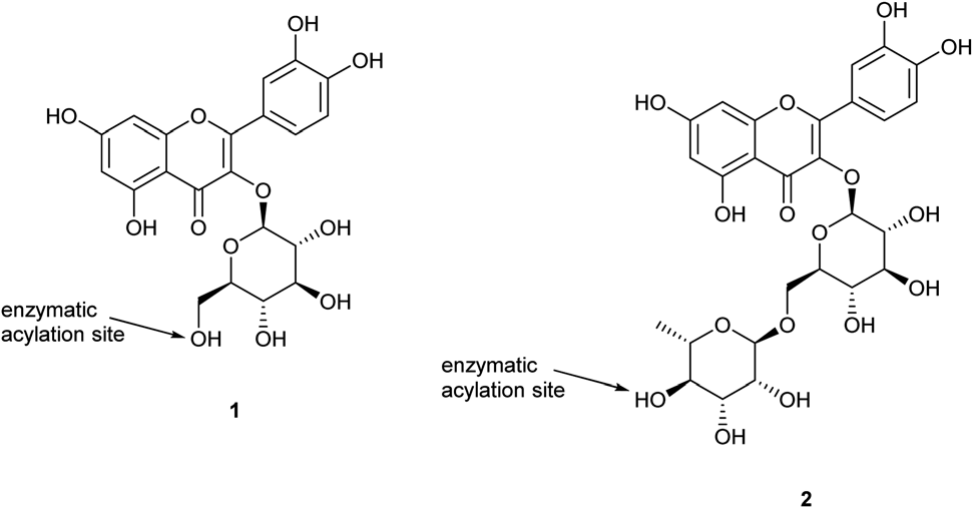


Despite the successful bioesterification work completed on flavonoid monosaccharides, there is limited research on selective acylation of flavonoid disaccharides. Rutin (quercetin-3-*O*-rutinoside) is one of these flavonoid disaccharides showing a range of bioeffects *in vitro* but with limited applications due to its low solubility in both water and oils (less than 0.10 mg g^−1^ and 0.25 mg g^−1^, respectively). Rutin also does not present any primary hydroxyl group, but only secondary hydroxyl groups, thus it is expected to be a challenging substrate for regioselectivity. Its wide distribution in the plant kingdom makes it a particularly attractive target compound for methodological development. Research on rutin (quercetin-3-*O*-rutinoside; 2) has shown evidence of acylation catalysed by *Ca*lB with palmitic acid,^2^ lauric acid,^2^ stearic acid,^21^ and unsaturated fatty acids,^4^ with regioselectivity of esterification specific at the C4′′′-position of the rhamnose moiety.

In most of the reported cases, reaction times longer than 96 hours were required, along with the use of molecular sieves to achieve the acylation of rutin in high yields. A major consideration in lipase-mediated bioesterifications is the choice of solvent, which has to solubilise both polar flavonoids and non-polar long-chain fatty acids; rutin has an experimentally determined log *P*_o/w_ of −0.64 ± 0.05, giving it low solubility in most enzyme-compatible organic media, in comparison with isoquercetin with log *P*_o/w_ of 0.76 ± 0.01.^22^

Herein, our aim was to establish an improved procedure for the enzyme-catalysed lipophilisation of rutin with a range of acyl donors of varying carbon chain length, investigating optimal reaction conditions (temperature, duration, agitation, enzyme, solvent) and examining the necessity of a dehydrating agent. The applicability of the acylation protocol on mixtures of compounds is also to be investigated as most natural products are found in nature in mixtures.

## 2. Results and discussion

### 2.1. Optimization of the reaction conditions

The desired acylation protocol was developed through reaction optimisation inspired by the work of Cruz *et al.*^15,16^ A series of variables were explored in acylation of rutin with *Ca*lB immobilized on Immobead 150, such as the acylating agent, the solvent system, and the dehydrating agent, along with temperature and duration. The conditions tested and the results obtained are presented in Table 1, where given conversion ratios are calculated by integrating peak areas of the parent compound and the acylated product as identified by LC-MS analysis of the crude reaction mixture after eliminating the solid particles and the excess of fatty acid (procedure described in detail in Section 4.9).

**Table 1:** Acylation of rutin with 20 g dm^−3^*Ca*lB immobilized on Immobead 150 under a variety of conditions

Exp. no.	Solvent	Conditions	Dehydrating agent	Fatty acid	Conversion ratio
1	10 : 1 acetone : DMSO	50 °C, 96 h	MS4Å (100 g dm^−3^)	Octanoic acid (100 eq.)	21%
2	10 : 1 acetone : DMSO	50 °C, 96 h	MS4Å (100 g dm^−3^)	Octanoic acid (10 eq.)	Nil
3	10 : 1 acetone : DMSO	50 °C, 96 h	MS4Å (100 g dm^−3^)	Octanoic acid (4 eq.)	Nil
4	10 : 1 acetone : DMSO	50 °C, 96 h	MS4Å (50 g dm^−3^)	Octanoic acid (100 eq.)	32%
5	10 : 1 acetone : DMSO	50 °C, 96 h	MS4Å (100 g dm^−3^)	Butyric acid (100 eq.)	24%
6	10 : 1 acetone : DMSO	50 °C, 96 h	MS4Å (100 g dm^−3^)	2-Methylbutyric acid (100 eq.)	Nil
7	Isopropanol	50 °C, 96 h	MS4Å (100 g dm^−3^)	Octanoic acid (100 eq.)	Nil
8	10 : 1 2-butanone : DMSO	50 °C, 96 h	MS4Å (100 g dm^−3^)	Octanoic acid (100 eq.)	Nil
9	Acetonitrile	55 °C, 48 h	MS4Å (100 g dm^−3^)	Octanoic acid (100 eq.)	34%
10	10 : 1 acetonitrile : DMSO	55 °C, 96 h	MS4Å (100 g dm^−3^)	Octanoic acid (100 eq.)	45%
11	10 : 1 acetonitrile : DMSO	55 °C, 96 h	MS4Å (100 g dm^−3^)	Octanoic acid (10 eq.)	34%
12	10 : 1 acetonitrile : DMSO	55 °C, 96 h	None	Octanoic acid (10 eq.)	20%
13	10 : 1 acetonitrile : DMSO	55 °C, 48 h	SiO_2_ (32.9 g dm^−3^)	Octanoic acid (100 eq.)	47%
14	10 : 1 acetonitrile : DMSO	55 °C, 96 h	MS4Å (100 g dm^−3^)	2-Methylbutyric acid (100 eq.)	Traces
15	10 : 1 acetonitrile : DMSO	55 °C, 96 h	MS4Å (100 g dm^−3^)	Palmitic acid (50 eq.)	Traces
16	10 : 1 acetonitrile : DMSO	55 °C, 96 h	MS4Å (100 g dm^−3^)	*p*-Coumaric acid (50 eq.)	Nil

Investigation into an appropriate solvent system was needed because solvents such as primary alcohols (*e.g.* methanol, ethanol) or water were not a viable option due to the nature of the reaction, despite rutin having good solubility in them; an easily accessible alcohol would lead to its preferential reaction with the fatty acid and the formation of the corresponding ester, whilst the ability of lipases to catalyse ester hydrolysis when water is present excluded water as a solvent option. A 10 : 1 mixture of acetone : DMSO dissolved rutin efficiently, but not fully, and did allow some acylation to occur at 50 °C (acetone b.p. 57 °C). The use of a secondary alcohol (isopropanol) did not yield any acylated product, nor did a 10 : 1 mixture of 2-butanone : DMSO. Although pure acetonitrile yielded positive acylation results, a 10 : 1 acetonitrile : DMSO mixture, as observed by Cruz *et al.* for anthocyanin glucosides,^15^ showed optimal results, and this solvent enabled an increase in reaction temperature to 55 °C (optimal temperature for *Ca*lB ranging between 45 and 60 °C). Monitoring of the reaction at 24, 48, 72 and 96 h showed that maximal substrate conversion was reached after 72 h, so the optimal duration was fixed at this time length. The data supporting this choice are presented in Fig. 2. An experiment was also conducted using a 9 : 2 acetonitrile : DMSO mixture (data not shown), but the conversion ratio was significantly lower, therefore other solvent mixtures were not explored further.

The results presented in Table 1 indicate that the optimal conditions for the acylation of rutin using *Ca*lB immobilized on the PMMA-based resin Immobead 150 involve acetonitrile : DMSO (10 : 1 ratio) as reaction solvent, 100 eq. of fatty acid, and heating at 55 °C for 72 h. Results obtained from analysis of reaction samples support these observations. One of the main findings that needed further clarification was the effect of SiO_2_, as its addition initially seemed to lead to full conversion of rutin to the corresponding acylated product, judging from the analysis of the aliquot collected at 48 h of reaction. Repetition of this experiment and analysis of the methanolic extract, after filtration of solid particles (see experimental procedure, Section 4.9), confirmed a slightly higher conversion ratio (47%) in comparison with experiments where MS4Å was used, but not full conversion of the substrate. Comparison of the two dehydrating agents also shows that MS4 Å has larger surface area (0.138 cm^3^) compared to SiO_2_ (0.061 cm^3^), and that the recovery of material from SiO_2_ is better. These observations led to further experiments using SiO_2_ as dehydrating agent instead of MS4Å (experiment 22 and on, unless stated differently).

**Fig. 1:**
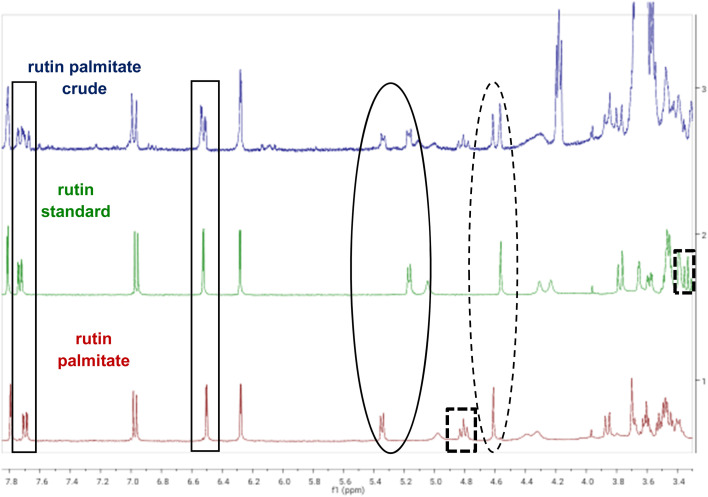
^1^H-NMR spectra of rutin (2) and the acylation reaction with palmitic acid in acetone-d_6_, yielding rutin palmitate (3d). Expansion 3.30–7.90 ppm: marked in rectangles are the shifts between the aromatic protons of rutin (5′ and 8) when the acyl ester is formed. Marked in the solid circle is the deshielded anomeric proton of glucose (1′′) and in the dashed circle is the deshielded anomeric proton of rhamnose (1′′′). Finally, the dashed squares include the shifts for proton 4′′′, which gets significantly deshielded when the ester is formed.

Table 1 also shows that carboxylic acid donors with shorter alkyl chains (butyric acid) have greater reactivity compared with fatty acids with longer alkyl chains (octanoic acid, palmitic acid). Additionally, it was observed that a bulkier α-substituted fatty acid (2-methylbutyric acid) can also react under the optimised conditions; in contrast, the reaction with a cinnamic carboxylic acid (coumaric acid) did not yield any acylated product. Typical characteristic changes in the ^1^H-NMR spectra for the ester products are shown in Fig. 1, where the shifts of the peaks corresponding to the aromatic protons of the flavone core and the anomeric protons of the two sugars can be seen. More specifically, small shifts are observed for protons 2′ (7.81 ppm for rutin *vs.* 7.79 ppm for the esters) and 6′ (7.73 ppm for rutin *vs.* 7.69 ppm for the esters). However, more significant shifts were recorded for the anomeric protons of the sugars which were deshielded in both cases. Therefore, the anomeric proton of glucose (1′′) appears at 5.33 ppm for the ester (instead of 5.17 ppm for rutin) whilst the anomeric proton of rhamnose (1′′′) appears as a singlet at 4.61 ppm (instead of 4.56 ppm for rutin). The most important observation though is the change occurring in the shift of proton 4′′′ which is indicative of the regioselective acylation of rutin on the hydroxy group of position 4′′′ in the rhamnose moiety. In the parent compound, the peak of this proton appears as a triplet at 3.33 ppm, but it is significantly deshielded for the esterified compound, appearing at 4.81 ppm. Additional details on the ^1^H-NMR spectra of the acylated compounds are presented in Table 7 as well as in Section 4.12 of the Experimental section.

Further experiments were necessary to complete the investigation of this protocol. As in all previous research done on acylation of rutin using *Ca*lB, the addition of a dehydrating agent seemed mandatory, one of the points that required clarification was the necessity of its presence in the reaction medium. For this reason, a rutin acylation experiment (exp. 12) was performed without using any means of dehydration, with octanoic acid in 10-fold excess; it was observed that the acylated compound was formed, although in a lower quantity. In order to monitor the production of the acylated compound by ^1^H-NMR, the aforementioned experiment (exp. 12) was repeated in deuterated solvents (acetonitrile and DMSO), again in the absence of dehydrating agent; aliquots of equivalent volumes (200 μL) were collected at *t*_0_ (addition of *Ca*lB), *t*_1_ (24 h) and *t*_4_ (96 h) and were diluted at 600 μL by adding deuterated acetonitrile. Although the obtained ^1^H-NMR data did not indicate any detectable production of water (peak of water in CD_3_CN appearing at 2.13 ppm), the spectra appeared identical, indicating that no observable amount of acylated product was formed in the experiment that took place in deuterated solvents. Therefore, it was concluded that the addition of a dehydrating agent is necessary to ensure ester formation was the dominant process.

A crucial factor in order to maximise acylation was lipase selection, so different enzymes were investigated, namely *Ca*lB immobilized on Immobead 150, *C. antarctica* lipase A (*Ca*lA) immobilized on Immobead 150, *Candida rugosa* lipase immobilized on beads, and *Ca*lB immobilized on acrylic resin. The choice of these enzymes was to examine activity differences between *C. antarctica* isoenzymes A and B that mainly differ on neutral sugar content, hydrophobicity, stability and substrate specificity,^23^ and to also afford a comparison with a third enzyme that was an unspecified isoenzyme of *Candida* lipase. It is clear from Table 2 that *Ca*lA afforded no acylation and, although *Candida rugosa* lipase and *Ca*lB both catalyse acylation, the latter one showed higher conversion. The effect of the nature of the immobilizing resin on the acylation was investigated by comparing the two different *Ca*lB enzymes and it was seen that when *Ca*lB was immobilized on acrylic resin it catalysed the esterification reaction of rutin more efficiently compared to the other enzymes used. Moreover, when the reactions were catalysed by enzymes immobilized on Immobead 150, a methacrylate polymer with epoxy function and particles' size varying between 150–300 μm, a specific signal was detected at 3.57 ppm in all NMR spectra of the crude reaction mixtures. This peak was absent in the spectra of reactions catalysed by enzymes immobilized on different resins and corresponds to the methoxy groups from the Immobead polymethylmethacrylate (PMMA) resin or its degradation products. This is probably observed due to the ability of certain solvents to dissolve part of PMMA; such solvents are the mixtures of acetonitrile/alcohol or CCl_4_/alcohol or alcohol/water.^24,25^ The enzyme of choice, *Ca*lB on acrylic resin, was commercially sourced, already immobilised on the macroporous acrylic polymer resin Lewatit VP OC 1600 *via* interfacial activation.^26^

**Table 2:** Acylation trials of rutin using different lipases with octanoic acid (100 eq.) in 10 : 1 acetonitrile : DMSO; 96 h, 55 °C, no mechanical stirring

Exp. no.	Enzyme (40 kU L^−1^)	Dehydrating agent	Conversion ratio
10	*Ca*lB on Immobead 150	MS4Å (100 g dm^−3^)	45%
16	*Ca*lA on Immobead 150	MS4Å (100 g dm^−3^)	Nil
17	*C. rugosa* lipase	MS4Å (100 g dm^−3^)	39%
18	*Ca*lB on acrylic resin	MS4Å (100 g dm^−3^)	46%
19	*Ca*lB on acrylic resin	SiO_2_ (32.9 g dm^−3^)	47%

In order to compare the two dehydrating agents, the most active enzyme was tested with both and SiO_2_ seemed to lead to slightly higher conversion ratios for rutin than when using MS4Å. Molecular sieves of pores size 4 Å are typically used as dehydrating agents as the zeolite porous material made of hydrated aluminosilicates is able to trap water molecules. However, we have also observed that the material readily interacts with flavonoids such as rutin *via* catechol–aluminium complexation, thus, unreacted rutin may accumulate around the molecular sieve beads/powder. Silicon dioxide potentially allows selective removal of water molecules formed during the esterification reaction without such interactions with rutin.

**Fig. 2:**
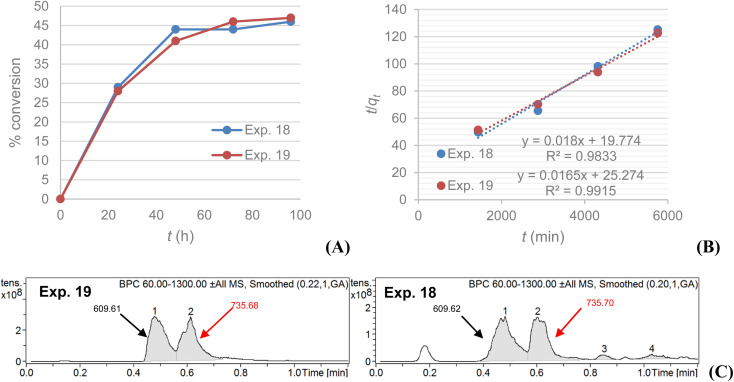
Experiments 18 (100 g dm^−3^ MS4Å) and 19 (32.9 g dm^−3^ SiO_2_): (A) % conversion of rutin to rutin octanoate product as monitored with LC-MS during the 96 h reactions; (B) plot of *t*/*q*_*t*_*vs. t* confirming a pseudo-second order conversion reaction; (C) LC-MS spectra comparison of the qualitative profile of the methanolic phases for each experiment; [M–H]^−^ shown (g mol^−1^).

A kinetic comparison between MS4Å (exp. 18) and SiO_2_ (exp. 19) on the acylation of rutin with octanoic acid, monitored by LC-MS, demonstrated that the reaction with SiO_2_ as dehydrating agent exhibits a slower, but steady, rate for conversion of rutin to rutin octanoate, as verified by LC-MS data and their statistical analysis (Fig. 2A). LC-MS chromatograms of the experiments also revealed that conversion using SiO_2_ afforded fewer, almost non-detectable, by-products in comparison with MS4Å; Fig. 2C shows that in exp. 19 only peaks for rutin (610.6 g mol^−1^) and rutin octanoate (736.6 g mol^−1^) are observed, whereas in exp. 18 additional peaks can be seen (peaks 3 and 4) attributed to as yet unidentified by-products, that correspond to approximately 10% of the contents of the crude reaction mixture, as calculated by integrating the corresponding NMR spectra.

The data were fitted to a pseudo-second order kinetic model as described in eqn (1):1
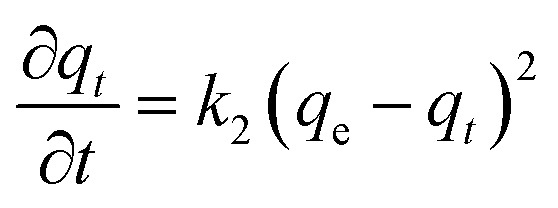
where *q*_*t*_ and *q*_e_ are % conversion at a given time (*t*) and at equilibrium, respectively. The term *k*_2_ is the rate constant of the pseudo-second order equation (% conversion min^−1^). For the boundary conditions *t* = 0 to *t* = *t* and *q*_*t*_ = 0 to *q*_*t*_ = *q*_*t*_, the integrated form of eqn (1) becomes eqn (2):2
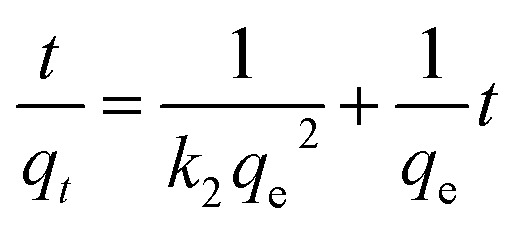


A plot of *t*/*q*_*t*_ against *t* that produces a straight line, confirms the model, where % conversion at equilibrium (*q*_e_) = 1/slope, and the pseudo-second order rate constant (*k*_2_) = slope^2^/intercept. Both experiments show high correlation (*R*^2^ > 0.98) with the pseudo-second order model (Fig. 2B). It is evident that MS4Å gives a slightly higher conversion rate (*k*_2_ = 1.64 × 10^−5^% min^−1^) in comparison with SiO_2_ (*k*_2_ = 1.08 × 10^−5^% min^−1^), but equilibrium conversion using MS4Å (*q*_e_ = 55.6%) is lower in comparison with SiO_2_ (*q*_e_ = 60.6%), but these results were statistically very close to each other.

A final verification experiment was also performed without addition of enzyme, to investigate the effect of silicon dioxide as catalyst in the formation of the acylated product; however, after 72 h of heating no product formation was observed. Given these results, all further experiments were performed with *Ca*lB immobilized on acrylic resin and SiO_2_ as dehydrating agent, unless stated differently. The quantity of SiO_2_ used was fixed at 32.9 g L^−1^ and no attempts were made to optimise this further.

It was demonstrated that the resin supporting the enzyme was not compatible with either mechanical stirring or sonication (Table 3), although there are literature references to using both techniques with resin-immobilized enzymes;^1,4,13,14^ the lack of scalability and the necessity of magnetic stirring also excluded the option of a microwave reactor. To maximize the contact surface of the enzyme with the reagents, a shaken water bath (exp. 22–25) was employed, but the results were not significantly improved compared to the previous experiments. The most promising outcome was observed from the use of a rotating wheel-like apparatus designed for solid phase synthetic chemistry (exp. 26), where similar conversion could be achieved at room temperature compared to previous experiments at higher temperatures, typically 55 °C. These results point to future use of flow systems to enhance the acylation process further, as the rotating wheel provides continuous mixing of the enzyme with the entity of the liquid phase without requiring the presence of a stirring bar – conditions that can be achieved in a flow system as well. In all cases the enzyme was used at a concentration of 40 kU L^−1^, but some literature suggested a concentration of 100 kU L^−1^ was optimal,^1,2^ so further research was conducted at this enzyme concentration (Table 4; exp. 31); the conversion ratio observed was similar to previous experiments (*e.g.*Table 2, entry 19). No experiments with non-immobilized lipase were conducted mainly due to the higher price of the free enzyme which, combined with the fact that a non-immobilized enzyme cannot be easily recovered, would lead to economically unfeasible process. In our protocol, the enzyme can be recovered but it is currently not possible to be reused due to the experimental procedure (see Section 4.9).

**Table 3:** Mechanically assisted trials of rutin acylation in 10 : 1 acetonitrile : DMSO with *Ca*lB

Exp. no.	Dehydrating agent	Fatty acid	Agitation and conditions	Conversion%
20	MS4Å (100 g dm^−3^)	Octanoic acid (100 eq.)	Magnetic stirrer bar, 55 °C, 24 h	Nil^a^
21	SiO_2_ (32.9 g dm^−3^)	Octanoic acid (100 eq.)	Ultrasonic, 45–60 °C, 2 h	Nil^a^
22	SiO_2_ (32.9 g dm^−3^)	Octanoic acid (100 eq.)	Shaking waterbath, 55 °C, 24 h	28%
23	SiO_2_ (32.9 g dm^−3^)	Octanoic acid (100 eq.)	Shaking waterbath, 55 °C, 72 h	28%
24	SiO_2_ (32.9 g dm^−3^)	Octanoic acid (10 eq.)	Shaking waterbath, 55 °C, 72 h	9%
25	SiO_2_ (32.9 g dm^−3^)	Palmitic acid (50 eq.)	Shaking waterbath, 55 °C, 72 h	Traces
26	SiO_2_ (32.9 g dm^−3^)	Octanoic acid (100 eq.)	Rotating wheel, r.t., 48 h	21%

a Resin (both Immobead 150 and acrylic resin) breakage caused by too much agitation.

**Table 4:** Trials of rutin acylation with 40 kU L^−1^*Ca*lB immobilized on acrylic resin; 72 h, 55 °C, no mechanical stirring

Exp. no.	Solvent	Dehydrating agent	Fatty acid	Conversion ratio
27	10 : 1 acetonitrile : DMSO	MS4Å (100 g dm^−3^)	Octanoic acid (10 eq.)	16%
28	10 : 1 acetonitrile : DMSO	MS4Å (100 g dm^−3^)	Palmitic acid (50 eq.)	15%
29	10 : 1 acetonitrile : DMSO	SiO_2_ (32.9 g dm^−3^)	Octanoic acid (10 eq.)	20%
30	10 : 1 acetonitrile : DMSO	SiO_2_ (32.9 g dm^−3^)	Octanoic acid (100 eq.)	38%^a^
31	10 : 1 acetonitrile : DMSO	SiO_2_ (32.9 g dm^−3^)	Octanoic acid (100 eq.)	45%^b^
32	10 : 1 2-butanone : DMSO	SiO_2_ (32.9 g dm^−3^)	Octanoic acid (100 eq.)	24%
33	10 : 1 acetonitrile : DMSO	SiO_2_ (32.9 g dm^−3^)	Acetic acid (100 eq.)	Traces^c^
34	10 : 1 acetonitrile : DMSO	SiO_2_ (32.9 g dm^−3^)	Butyric acid (100 eq.)	30%
35	10 : 1 acetonitrile : DMSO	SiO_2_ (32.9 g dm^−3^)	2-Methylbutyric acid (100 eq.)	7%
36	10 : 1 acetonitrile : DMSO	SiO_2_ (32.9 g dm^−3^)	Palmitic acid (50 eq.)	19%
37	10 : 1 acetonitrile : DMSO	SiO_2_ (32.9 g dm^−3^)	Stearic acid (50 eq.)	8%
38	10 : 1 acetonitrile : DMSO	SiO_2_ (32.9 g dm^−3^)	Oleic acid (50 eq.)	15%
39	10 : 1 acetonitrile : DMSO	SiO_2_ (32.9 g dm^−3^)	Methyl oleate (50 eq.)	15%
40	10 : 1 acetonitrile : DMSO	MS4Å (100 g dm^−3^)	*p*-Coumaric acid (10 eq.)	Nil
41	10 : 1 acetonitrile : DMSO	SiO_2_ (32.9 g dm^−3^)	Lauric acid (50 eq.)	30%

a Total reaction volume was 5 mL.

b 100 kU L^−1^*Ca*lB on acrylic resin.

c Evidence of non-selective acylation.

The nature and concentration of the fatty acid acyl donor was investigated (Table 4) and it was observed that donors with short alkyl chains (butyric acid; C_4_) and medium alkyl chains (octanoic and lauric acid; C_8_, C_12_) react more easily than donors with longer alkyl chains (C_16_, C_18_, C_18:1_). The reactivity of longer chain fatty acids depends both on the length of the chain and the solubility of the fatty acid in the reaction medium, as noted from the lower reaction yield for stearic acid (C_18_) in comparison with its unsaturated counterpart oleic acid (C_18:1(*cis*)_). A bulkier α-substituted fatty acid (2-methylbutyric acid; exp. 35) also showed evidence of reaction, but in lower yields, whereas the reaction with an aromatic carboxylic acid (*p*-coumaric acid; exp. 40) did not lead to any acylated product. Although the use of 100 eq. of fatty acid has proven to be very efficient, acylation occurred also when only 10 eq. of fatty acid was added but the conversion ratios were lower. This is a result that needs to be explored further in the future to make the whole procedure more sustainable.

The use of esters as acyl donors instead of fatty acids (methyl oleate; exp. 39) also led to acylation with similar conversion ratios compared to the corresponding fatty acids. This observation suggests that the idea of transesterification reactions should be further examined in the future as it can help us reduce or even eliminate the amount of dehydrating agent used, since water will no longer be a by-product of the procedure. Experiments with different fatty acids were repeated under both the initial and the optimised reaction conditions (SiO_2_ instead of MS4Å; *Ca*lB immobilized on acrylic resin instead of Immobead 150) and, although the conversion ratio remained moderate, in all cases it was higher compared to the previous efforts. Future research will apply the esterification protocol on other fatty acids such as small dicarboxylic acids and longer alkyl chain fatty acids.

### 2.2. Acylation of model flavonoid mixture compositions

As mentioned earlier, we required the developed acylation protocol to be applicable to mixtures of compounds, since most natural products are found in nature in mixtures and are used in this form in commercial applications. A successful protocol would allow the application of this method on mixtures of flavonoids related to ongoing research on extracts from the skins of blackcurrant (*Ribes nigrum*)^27^ and aronia (*Aronia melanocarpa*).^28^ In those extracts, various flavonoid glycosides coexist; therefore, protocol efficacy was examined on mixtures of glucosides and rutinosides with experiments performed under both the initial and the optimized reaction conditions, designed specifically for this purpose. As seen in Table 5, both isoquercetin and rutin are converted to the corresponding acylated compounds; however, the conversion ratio could not be determined for each one of the starting materials separately because in both LC-MS and UHPLC-UV analyses the two glycosides elute under the same peak, as do their acylated counterparts. ^1^H-NMR spectra indicate acylation of both compounds as well. An estimation for the individual conversion ratios of the two compounds can be obtained by comparing the chromatograms of exp. 44 and 45. In exp. 44 amounts of equal weight were used for the two substrates, leading to an isoquercetin : rutin molar ratio of 1 : 0.8; in exp. 45 the amounts used were equimolar. The conversion ratios calculated by the corresponding chromatograms seem to confirm the easier acylation of the glucoside compared to rutinoside, as the conversion percentage is higher for the experiment that uses higher amount of isoquercetin as substrate.

**Table 5:** Acylation trials of mixtures with octanoic acid (100 eq.) in 10 : 1 acetonitrile : DMSO; 72 h, 55 °C, no mechanical stirring

Exp. no.	Substrate	Enzyme (40 kU L^−1^)	Dehydrating agent	Conversion ratio
41	Isoquercetin	*Ca*lB on Immobead 150	MS4Å (100 g dm^−3^)	80%
42	Rutin + isoquercetin	*Ca*lB on Immobead 150	MS4Å (100 g dm^−3^)	77%
43	Rutin + isoquercetin	*Ca*lB on acrylic resin	MS4Å (100 g dm^−3^)	83%
44	Rutin + isoquercetin	*Ca*lB on acrylic resin	SiO_2_ (32.9 g dm^−3^)	88%
45	Rutin + isoquercetin	*Ca*lB on acrylic resin	SiO_2_ (32.9 g dm^−3^)	62%

In general, the conversion ratios obtained in the experiments of the mixtures are increased compared to the ones recorded for rutin. This can be explained by the presence of isoquercetin in the mixture and the significantly higher reactivity possessed by the free primary alcohol group present on the glucose moiety of the monosaccharide. It is also clearly indicated by the higher conversion ratio observed for isoquercetin itself in exp. 41.

### 2.3. Synthesis and isolation of rutin derivatives

Synthesis of several representative compounds was repeated on a larger scale under the optimised reaction conditions, specifically rutin butyrate (3a), rutin octanoate (3b), rutin laureate (3c), rutin palmitate (3d), rutin stearate (3e), and rutin oleate (3f). Purification was achieved using preparative Reversed Phase High Pressure Liquid Chromatography (prep. RP-HPLC); separation of the compounds was satisfactory, enabling more accurate analytical data (LC-MS, NMR spectra) to be obtained. However, this still only provided very limited quantities of sample for more extensive analysis. Hence a different technique was enabled, the Reversed Phase Medium Pressure Liquid Chromatography (RP-MPLC). This technique was successfully applied to quantities varying from few milligrams up to grams simply by changing the size of the column-cartridge used for the separation; a Biotage® system was used as described in Section 4.6.
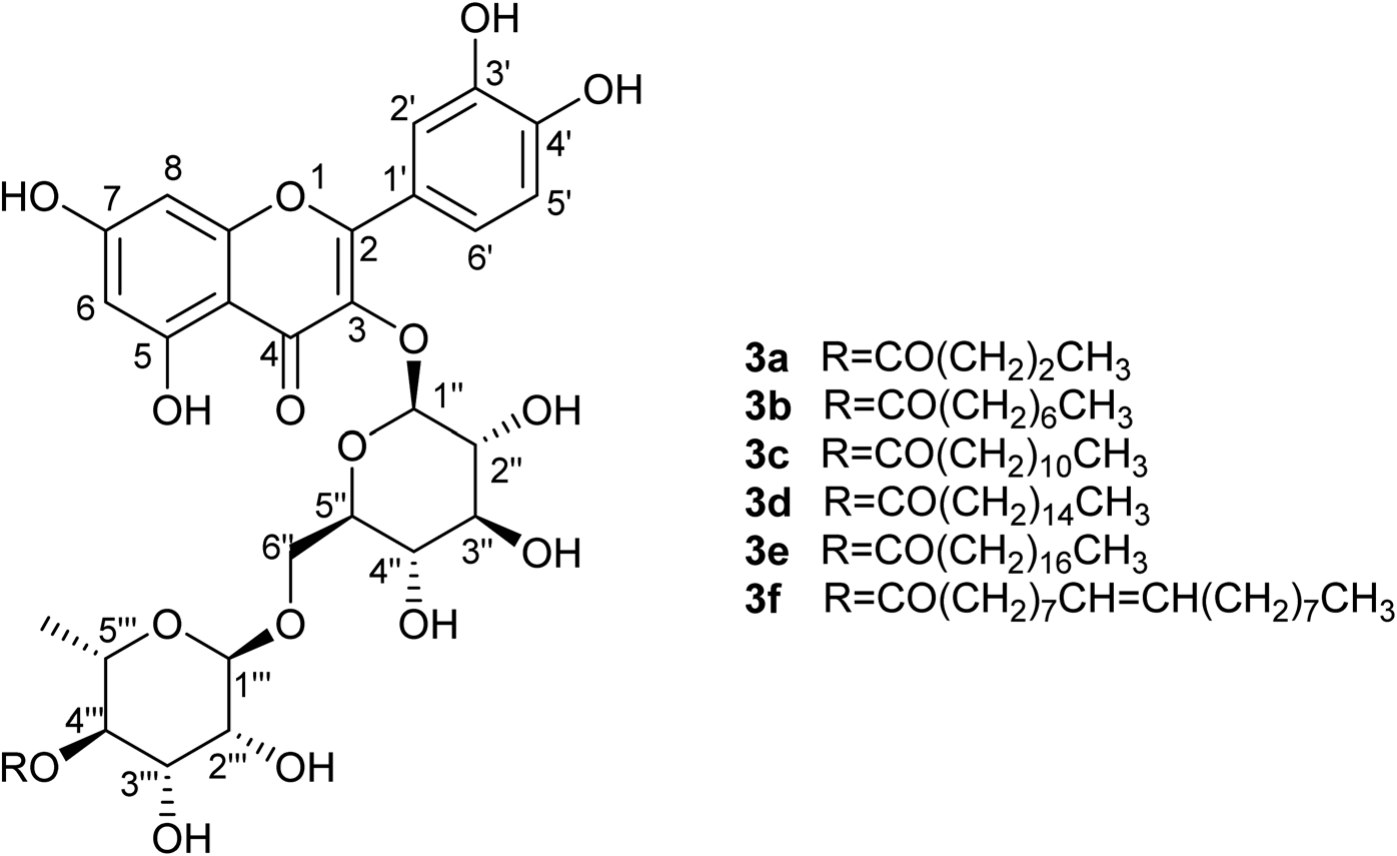


**Fig. 3:**
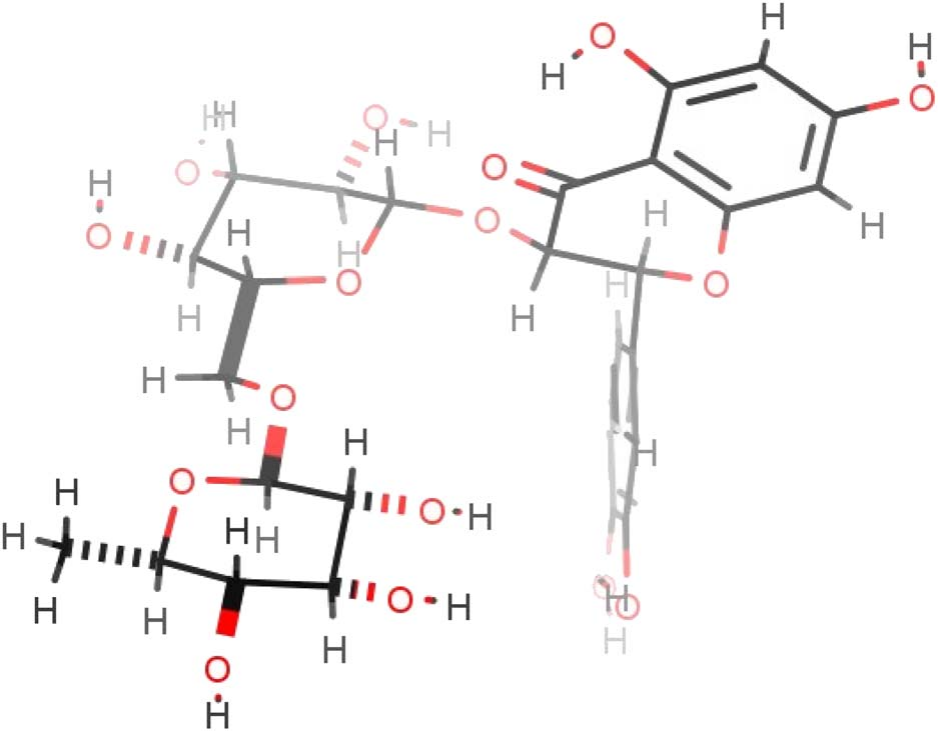
The most energetically favourable conformation of rutin (2) (509.8 kJ mol^−1^), as predicted by ChemAxon Marvin Sketch.

Addition of alkyl chains offers a significant difference in the polarity between the reactant and the acylated products and enabled effective separation of the compounds. Yields are presented in Table 7 and are calculated based on the MPLC-isolated quantity of the acylated products and are comparable to the previously given conversion ratios from LC-MS analysis. Some spectroscopic data for the isolated compounds are presented in Table 6 confirming the structures and demonstrating that the acylation is selective at the 4-OH position of the rhamnopyranosyl moiety on the disaccharide unit (details in Experimental). This suggestion is also supported by bibliographic data^2,21^ and by the three-dimensional conformation of rutin (2; Fig. 3), which demonstrates that the hydroxyl group in position 4′′′ is the most easily accessible one for acylation compared to the other hydroxyl groups present on the molecule. These observations are supported by the spectroscopic data obtained; significant change is observed in the chemical shifts of all rhamnose protons but, as already analysed, the most important difference occurs for the shift of proton 4′′′ (at 4.81 ppm instead of 3.33 ppm for rutin), indicative of the acylating position. Additionally, both protons 3′′′ and 5′′′ are significantly deshielded along with the methyl group of the sugar.

**Table 6:** ^1^H NMR spectroscopic data for the synthesized compounds in acetone-d_6_

Atom	Rutin (2)	Rutin butyrate (3a)	Rutin octanoate (3b)	Rutin laurate (3c)	Rutin palmitate (3d)	Rutin stearate (3e)	Rutin oleate (3f)
	*δ*_H_ (ppm), *J* (Hz)	*δ*_H_ (ppm), *J* (Hz)	*δ*_H_ (ppm), *J* (Hz)	*δ*_H_ (ppm), *J* (Hz)	*δ*_H_ (ppm), *J* (Hz)	*δ*_H_ (ppm), *J* (Hz)	*δ*_H_ (ppm), *J* (Hz)
5	12.40 bs	12.47 bs	12.44 bs	12.45 bs	12.47 bs	12.47 bs	12.47 bs
6	6.28 d (2.1)	6.28 d (2.0)	6.27 d (2.1)	6.27 d (2.1)	6.27 d (2.1)	6.28 d (2.0)	6.28 d (1.9)
8	6.52 d (2.1)	6.50 d (2.0)	6.51 d (2.1)	6.50 d (2.1)	6.50 d (2.1)	6.50 d (2.0)	6.50 d (1.9)
2′	7.81 d (2.2)	7.80 d (2.1)	7.79 d (2.2)	7.78 d (2.1)	7.78 d (2.1)	7.80 d (2.1)	7.80 d (2.1)
5′	6.97 d (8.5)	6.97 d (8.5)	6.97 d (8.5)	6.97 d (8.5)	6.97 d (8.5)	6.98 d (8.5)	6.98 d (8.4)
6′	7.73 dd (8.5, 2.2)	7.71 dd (8.5, 2.1)	7.69 dd (8.5, 2.2)	7.70 dd (8.5, 2.1)	7.70 dd (8.5, 2.1)	7.69 dd (8.5, 2.1)	7.70 dd (8.4, 2.1)
1′′	5.17 d (7.6)	5.36 d (7.2)	5.33 d (7.2)	5.34 d (7.3)	5.34 d (7.3)	5.35 d (7.2)	5.35 d (7.0)
2′′	3.45 m	3.48 m	3.48 m	3.47 m	3.47 m	3.47 m	3.45 m
3′′	3.48 m	3.48 m	3.48 m	3.47 m	3.47 m	3.49 m	3.48 m
4′′	3.39 m	3.39 m	3.38 t (9.0)	3.38 m	3.38 m	3.38 m	3.38 t (8.7)
5′′	3.39 m	3.43 m	3.42 dd (5.7, 1.3)	3.42 dd (5.7, 1.1)	3.42 dd (5.6, 1.2)	3.43 dd (5.8, 1.2)	3.43 dd (5.7, 1.1)
6′′α	3.78 d (11.4)	3.87 dd (10.9, 1.0)	3.85 dd (10.8, 1.3)	3.85 dd (10.7, 1.1)	3.85 dd (10.9, 1.2)	3.87 dd (11.0, 1.2)	3.87 dd (10.9, 1.1)
6′′β	3.45 m	3.52 m	3.51 dd (10.8, 5.7)	3.51 dd (10.7, 5.7)	3.51 dd (10.9, 5.6)	3.51 dd (11.0, 5.8)	3.52 dd (10.9, 5.7)
1′′′	4.56 s	4.62 s	4.61 s	4.61 s	4.61 s	4.61 s	4.61 s
2′′′	3.65 dd (3.3, 1.4)	3.70 bs	3.69 bs	3.70 bs	3.70 bs	3.69 bs	3.70 bs
3′′′	3.58 dd (9.3, 3.3)	3.69 m	3.68 dd (9.5, 3.4)	3.68 dd (9.5, 3.3)	3.68 dd (9.5, 3.2)	3.68 dd (9.4, 3.6)	3.67 dd (9.5, 3.3)
4′′′	3.33 t (9.3)	4.81 t (9.2)	4.81 t (9.5)	4.81 t (9.5)	4.81 t (9.5)	4.81 t (9.4)	4.81 t (9.5)
5′′′	3.48 m	3.62 m	3.61 dq (9.5, 6.3)	3.60 dq (9.5, 6.3)	3.60 dq (9.5, 6.3)	3.60 dq (9.4, 6.2)	3.61 dq (9.5, 6.2)
–CH_3_ (rha)	1.10 d (6.2)	0.91 m	0.92 d (6.3)	0.92 d (6.3)	0.92 d (6.3)	0.92 d (6.2)	0.92 d (6.2)
–CH_2_αC=O	—	2.24 td (7.4)	2.27 td (7.4, 3.2)	2.27 td (7.3, 3.2)	2.27 td (7.5, 3.2)	2.27 t (7.1)	2.27 t (7.4)
–CH_2_βC=O	—	1.57 sext (7.4)	1.56 m	1.55 m	1.56 m	1.55 m	1.59 quint (7.1)
–CH_2_ (FA)	—	—	1.29 bs	1.28 bs	1.28 bs	1.28 bs	1.30 bs
–CH_3_ (FA)	—	0.90 t (6.9)	0.87 t (6.9)	0.86 t (6.8)	0.87 t (6.7)	0.87 t (6.7)	0.88 t (6.8)
–CH=CH–	—	—	—	—	—	—	5.34 m
–CH_2_αCH=	—	—	—	—	—	—	2.08 bs

The highest yield is achieved for the medium-length chain liquid octanoic acid, thereafter, yield decreases with increase in the length of the alkyl chain. This difference is most likely caused by the decreasing solubility of the fatty acids in the reaction medium that occurs with longer alkyl chains. A notable difference is observed in both the conversion ratio and the yield for the stearic (C_18_) and oleic acid (C_18:1(*cis*)_) derivatives, the latter providing double the yield of its saturated counterpart, most likely due to its liquid nature affording greater miscibility with the reaction medium.

### 2.4. Evaluation of lipophilicity and antioxidant capacity

**Fig. 4:**
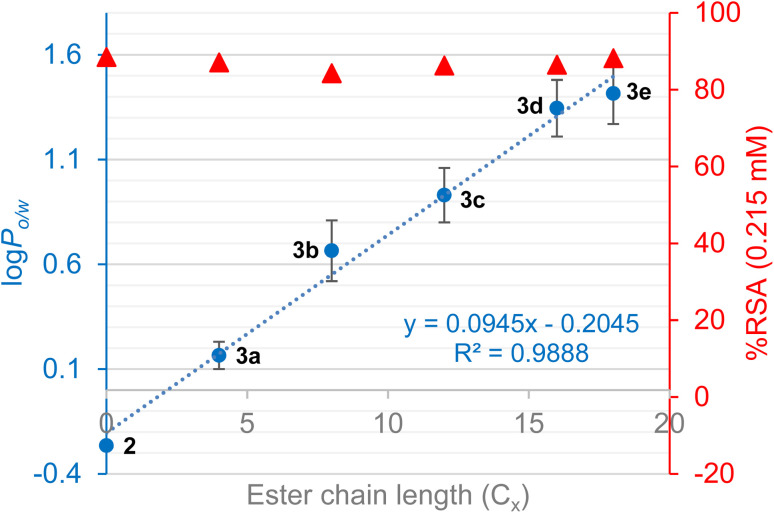
Log *P*_o/w_ (average of two measurement methods; error bars show SD between methods) and % RSA for rutin and rutin esters with increasing alkyl chain length (only saturated alkyl chains).

Investigation of the physicochemical and antioxidant properties of the rutin esters shows that lipophilicity (log *P*_o/w_) increases with increasing alkyl chain length, except for the unsaturated oleic acid (Table 7). It is noteworthy that the concentration of solutions prepared for calibration curves became lower as the length of the alkyl chain increased due to the reduced solubility of the ester in the methanol–water mixture. It was also observed that most rutin esters are excellent emulsifiers due to the presence of both hydrophilic and lipophilic moieties on the same molecule, with several hours often required to achieve complete separation of the two phases for the measurement of log *P*_o/w_. Further comparison of rutin and rutin esters (saturated alkyl chains only) is shown in Fig. 3 where it was observed that average log *P*_o/w_, taken across the two measurement methods used, increased linearly (*R*^2^ = 0.9888) with increasing alkyl chain length (*C*_*x*_) of the ester derivative (log *P*_o/w_ = 0.095*C*_*x*_ − 0.205). Evaluation of antioxidant activity (Table 7; Fig. 4) demonstrated that the rutin esters retained the pharmacological properties of the parent compound and % RSA was unaffected by increasing alkyl chain length of the ester; in combination with the observed increased lipophilicity, this makes these derivatives interesting candidates for incorporation in novel cosmetics and food products.

**Table 7:** Calculation of reaction yield, log *P*_o/w_ and % RSA values for esterification products

Compound	Ester chain	[M + H]^+^^a^ (g mol^−1^)	Yield	Log *P*_o/w_ (method A)	Log *P*_o/w_ (method B)	Log *P*_o/w_ (calculated)^b^	% RSA (0.125 mM)
Rutin (2)	—	611.66	—	−0.26	−0.27	−2.05	88.5
Rutin butyrate (3a)	C_4_	681.47	23%	0.10	0.23	−0.59	87.1
Rutin octanoate (3b)	C_8_	737.53	47%	0.52	0.81	0.99	84.3
Rutin laureate (3c)	C_12_	793.25	41%	0.80	1.06	2.58	86.3
Rutin palmitate (3d)	C_16_	849.49	33%	1.21	1.48	4.16	86.5
Rutin stearate (3e)	C_18_	877.40	10%	1.27	1.56	4.95	88.2
Rutin oleate (3f)	C_18:1(*cis*)_	875.18	27%	1.18	1.43	4.69	84.9

a Molecular ion as found by LC-MS analysis.

b Method for log *P* prediction developed by ChemAxon and is based on the method of Viswanadhan *et al*.^29^

### 2.5. Optimized scalable protocol

In order to make this newly developed protocol more attractive for larger scale preparation, some of the main defects were addressed. One issue concerned the low concentration of the substrate in the reaction medium (4.4 mM); a second issue related to the excessive amount of acylating agent required for obtaining the optimal reaction results (100 eq.). Accordingly, the substrate concentration was tripled; the reactions were again monitored with LC-MS which showed the conversion ratios were significantly higher than the ones previously recorded, reaching 80%, despite the substrate not being fully soluble in the reaction medium. More experiments were conducted to examine the maximum substrate concentration that can lead to increased conversion ratios, with the results indicating that concentrations as high as 22.2 mM do not improve the reaction profile (conversion ratio ∼67%).

**Fig. 5:**
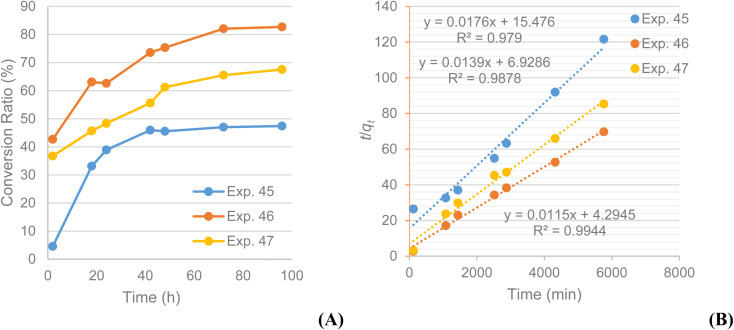
Experiments 45 ([S] = 4.44 mM), 46 ([S] = 13.3 mM) and 47 ([S] = 22.2 mM): (A) % conversion of rutin (2) to rutin octanoate (3b) product as monitored with LC-MS during the 96 h reactions; (B) plot of *t*/*q*_*t*_*vs. t* confirming a pseudo-second order conversion reaction.

To clarify any difference in the reaction kinetics for the various substrate concentrations, kinetics experiments were performed over 96 h as showed in Table 8. Samples were analysed at 2, 18, 24, 42, 48, 72 and 96 h with LC-MS. The conversion ratios over time were plotted against time and presented in Fig. 5A, which suggests that the median concentration (13.3 mM) leads to the highest conversion for the substrate under these conditions.

**Table 8:** Conditions for the kinetics experiments of rutin acylation in various concentrations by *Ca*lB on acrylic resin with octanoic acid in 10 : 1 acetonitrile : DMSO; 55 °C, no mechanical stirring

Exp. no.	Rutin concentration (mM)	Reaction time (h)	Octanoic acid equivalents	Maximum conversion ratio
44	13.3	72	100	82%
45	4.4	96	100	58%
46	13.3	96	100	82%
47	22.2	96	100	67%
48	13.3	48	10	53%

The same pseudo-second order kinetics model was also applied to these data, and from the graphs obtained it became obvious that it is the appropriate one for these kinetics data too. The plots of *t*/*q*_*t*_ against *t* produce straight lines as for the previous cases, confirming the model whilst % conversion at equilibrium (*q*_e_) and the pseudo-second order rate constant *k*_2_ were calculated as well. All experiments show high correlation (*R*^2^ > 0.98) with the pseudo-second order model (Fig. 5B). It is evident that the experiment conducted at a 13.3 mM concentration has the highest conversion rate (*k*_2(13.3)_ = 3.08 × 10^−5^% min^−1^) compared to the other two (*k*_2(4.4)_ = 2.00 × 10^−5^% min^−1^; *k*_2(22.2)_ = 2.79 × 10^−5^% min^−1^). The same is valid for the equilibrium conversion (*q*_e_), which is 87.0% for the experiment at 13.3 mM and lower for the experiments at 4.4 mM (56.8%) and 22.2 mM (71.9%). The calculated constants for the pseudo-second order kinetics model confirm the initial assumptions that the experiment conducted at a substrate concentration 3 times than the original one has faster product formation rate and yields larger product quantities. The encouraging results from these trials led to considering a reduction in the large excess of acylating agent required. Repeating the reaction at the newly optimised concentration but with a 10-fold excess of acylating agent showed a conversion ratio after 48 h of 53% (exp. 48), significantly higher than the 20% conversion observed for the corresponding experiment (exp. 28). The LC-MS chromatograms for experiments 45, 46 and 47 are included in the ESI.†

## 3. Conclusions

This research investigated the enzymatic esterification of natural compounds by *Candida antarctica* lipase B, and the physicochemical and antioxidant properties of the product esters. Evidence for the optimal reaction conditions were collected from the data obtained and indicate the use of an immobilized enzyme on acrylic resin in an optimal concentration of 40 kU L^−1^, which affords acylation of flavonoid glycosides even in mixtures. The ideal solvent system and conditions were demonstrated to comprise acetonitrile and DMSO in a 10 : 1 mixture, with reaction at 55 °C for 72 h. Optimal results were achieved when the substrate concentration rose to 13.3 mM whilst the presence of a dehydrating agent in the reaction seems mandatory, with the most suitable one being SiO_2_. The fatty acid was best added in 10-fold excess and the reaction was successful and regioselective for fatty acids bearing alkyl chains from 4 to 18 carbon atoms long but not for aromatic carboxylic acids. The lipophilicity of the rutin esters produced increased with increasing alkyl chain length, yet antioxidant properties were unaffected in comparison with the parent compound. The optimized protocol is economically manageable, easily applicable, and potentially scalable. Although several steps are still needed towards the development of an environmentally benign and industrially suitable protocol, the current methodology provided an insight into the reaction's key features and a better understanding of the product properties. This research offered the opportunity to synthesise natural products analogues that can find manifold industrial applications in the future.

## 4. Experimental part

### 4.1. Materials

The following commercially available *C. antarctica* lipases were used for the experiments: lipase B immobilized on Immobead 150, recombinant from yeast with activity 2000 propyl laurate units (PLU) per gram; lipase A immobilized on Immobead 150, recombinant from *Aspergillus oryzae* with activity of 500 PLU g^−1^; lipase immobilized on beads with activity of 2000 PLU g^−1^; and lipase B immobilized on acrylic resin, recombinant from *Aspergillus niger* with activity 5000 PLU g^−1^, formerly referred as Novozym^®^ 435. As all enzymes were commercially sourced, all specifications about immobilization and uniformity are in accordance with manufacturers' specifications, but detail of these are not provided by the supplier (Sigma-Aldrich). Molecular sieves 4 Å (MS4Å) were activated overnight in an oven at 150 °C. Silicon dioxide (SiO_2_) was supplied as silica gel 60 (40–60 μm), which can absorb up to 40% of its weight in water. All enzymes, solvents (99%+ purity), MS4Å, SiO_2_, fatty acids (97%+), rutin hydrate (95%), isoquercetin (>90%), and all general-purpose chemicals were purchased from Sigma-Aldrich Ltd, Dorset, UK. Water employed for HPLC analysis was obtained from a Milli-Q (Millipore, Milford, MA, USA) water purification system.

### 4.2. LC-MS

Liquid Chromatography with Mass Spectrometry (LC-MS) was carried out at room temperature on a Phenomenex Kinetex C18 column, 2.6 μm particle size, 50 × 2.1 mm I.D. column. Chromatography was carried out using two solvents: (A) water and 0.1% formic acid solution and (B) acetonitrile and 0.1% formic acid solution. A linear gradient programme was applied: 2–95% increase of solvent B over 1 min. The flow rate during the experiment was 1.3 mL min^−1^. Injections were made by a Basic Marathon autosampler equipped with a 20 μL loop. The method was carried out on a Thermo Ultimate 3000 UHPLC using a Bruker Amazon Speed IonTtrap for the MS detection and a Diode Array Detector. The ESI (electrospray ionisation) parameters in the negative ion mode were as follows: spray voltage 4000 V (applied to the spray tip needle), dry gas 10 dm^3^ min^−1^, dry temperature 365 °C, capillary 60 nA, nebulizer 65 psi, nebulising gas N_2_.

### 4.3. NMR

Nuclear magnetic resonance (NMR) spectra were recorded at 400 MHz on a Bruker Avance III HD-400 (AV400) spectrometer. Chemical shifts are reported in parts per million (ppm) downfield of tetramethylsilane (TMS) for proton resonances or residual solvent peaks; the samples were dissolved in (CD_3_)_2_CO (deuterated acetone), with reference peaks at 2.05 and 29.84 for ^1^H and ^13^C, respectively. The proton coupling constants are corrected and given in Hz and expressed as multiplicities, singlet (s), broad singlet (bs), doublet (d), doublet of doublets (dd), triplet (t), triplet of doublets (td), triplet of doublet of doublets (tdd), quartet (q), doublet of quartets (dq), quintet (quint) and multiplet (m). To aid characterisation, 2-D heteronuclear single quantum coherence (^1^H–^13^C HSQC), heteronuclear multiple bond correlation (^1^H–^13^C HMBC) and double quantum filtered correlation (^1^H–^1^H COSY) were utilised.

### 4.4. Analytical HPLC

The analytical HPLC system was an Agilent 1290 infinity series, equipped with a diode-array detector (DAD), a binary pump system connected with online degasser and Zorbax Eclipse XDB C18, 150 × 4.6 mm, 5 μm. The binary elution solvent system consisted of water and acetonitrile, both acidified with 0.5% v/v trifluoroacetic acid and aliquots of 20 μL of the samples, dissolved in acetonitrile, were injected with MicroAutosampler (Agilent 1200 series). The elution was gradient from 5% to 20% acetonitrile in the first 20 min, then increase to 100% acetonitrile within 3 min followed by isocratic elution (100% acetonitrile) for 1 min, and then linear decrease to 5% acetonitrile in 1 min followed by 5% acetonitrile isocratic elution for 5 more minutes. The flow rate was 1 mL min^−1^ and the UV detector monitored at 254, 285, 325, 350, and 520 nm.

### 4.5. UV/visible spectrophotometry

The spectra required for the calculation of compounds' lipophilicity were recorded in a Jasco V-630 spectrophotometer in the full UV-visible range of the spectrum (200–800 nm), at 1 nm intervals. For the evaluation of the antioxidant activity of the compounds a JenWay 6300 spectrophotometer and the absorption of the solutions was measured at one of the maximum absorption wavelengths (517 nm).

### 4.6. RP-MPLC (Biotage)

The isolation of the synthesized compounds was performed with Reversed Phase Medium Pressure Liquid Chromatography (RP-MPLC) using a Biotage-Isolera Prime. The column was a 12 g Biotage SNAP Ultra C18 eluted with acetonitrile and water, acidified with 0.1% formic acid. The elution started as isocratic at 5% acetonitrile for 1 column volume, with a gradient from 5% to 100% acetonitrile for 10 column volumes, and then isocratic at 100% for 2 column volumes.

### 4.7. Octanol-water partition coefficient determination

To evaluate the lipophilicity of the synthesized compounds, partition coefficient between octanol and water (log *P*_o/w_) was determined using two different methods, aiming to provide the strongest verification of the results obtained. In the first procedure, a 0.4 mM stock solution of the analyte (purity >90% as determined by LC-MS) was prepared in methanol, which was subsequently diluted to half its' concentration by the addition of an equivalent volume of distilled water. Four further dilutions were then performed for each compound with methanol and water in a 1 : 1 ratio as solvent. A calibration curve expressing the concentration–absorbance relationship was then constructed for each compound by measuring the absorbance of each solution at *λ*_max_ (291 nm). In the next step, 10 mL of a 0.1 mM aqueous solution of each compound were transferred to a separating funnel, then 10 mL of octanol were added, and the funnel was shaken vigorously for 30 s. The emulsion was left to settle for as much time as required to achieve full separation of the two phases. Notably, for the esters bearing longer alkyl chains, the separation of two phases required an overnight procedure. An aliquot of 1 mL was collected from the aqueous layer to which 1 mL of methanol was added, resulting in solutions with the same solvent mixture as that used for calibration curves. Absorbance of the solution was then measured at 291 nm and the concentration of the compound in the water phase (*C*_w_) was calculated from the equations of the corresponding calibration curves. The concentration of the compound in the octanol layer (*C*_o_) was calculated by subtracting *C*_w_ from the initial compound concentration pre-partitioning and the log *P*_o/w_ was subsequently calculated according to eqn (3):3
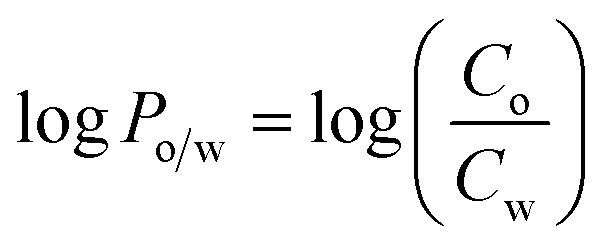


The second procedure was based on a method described by Rothwell *et al.*^22^ using a centrifuge instead of the shaken flask method. For this method, 0.2 mM solutions of each ester were prepared in methanol and aliquots placed in 1.5 mL Eppendorf vials (200 μL). The methanol was evaporated by air-drying and the residue left was dissolved by vigorous mixing in 200 μL of either 1-octanol or Tris–HCl buffer (pH 7.4), depending on the solubility of each compound, reaching a final concentration of 0.2 mM. The remaining phase was then added, and each replicate was further mixed. The two phases were centrifuged using a FisherBrand Benchtop High-Speed Mini-Centrifuge (5 min; 2900 g). Following separation, both layers were transferred in different vials and an aliquot of 100 μL was collected and diluted with 200 μL of methanol. Both samples were submitted to HPLC analysis with the same method described earlier and the area of the HPLC peaks of the compound, both in octanol and in the buffer, was measured. Lipophilicity was again determined by the partition coefficient (log *P*_o/w_); theoretical log *P*_o/w_ values were also calculated using MarvinSketch software (ChemAxon). The combined results are presented in Table 7.

### 4.8. Radical scavenging activity

For the evaluation of the antioxidant activity, radical scavenging activity (% RScA) was determined by investigating the ability of the compounds in various concentrations to reduce the DPPH radical (2,2-diphenyl-1-picrylhydrazyl), following a protocol described by Guldbrandsen *et al.*^30^ A solution of DPPH (16.5 mg in 300 mL ethanol) was prepared first and then the solutions of the synthesized compounds in methanol, in a concentration range from 0.008 to 1.0 mM. The experiments were performed in triplicate, the absorbance of four different solutions was measured at 517 nm after incubation for 30 min in the dark, and the activity was calculated using eqn (4):4
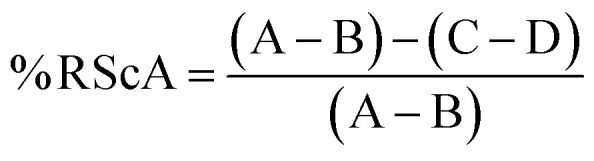
where A is a mixture of 1.0 mL of DPPH solution and 1.0 mL of methanol, B is a mixture of 1.0 mL of ethanol and 1.0 mL of methanol, C is a mixture of 1.0 mL of DPPH solution and 1.0 mL of test compound solution, and D is a mixture of 1.0 mL of ethanol and 1.0 mL of test compound solution. The representative concentration 0.125 mM was chosen to be presented in Table 7.

### 4.9. Initial methodology of the reaction

Activated MS4Å (739 mg) were added to a solvent mixture of 10 : 1 acetone : DMSO (7.39 mL) and stirred for 30 min to remove residual moisture. The substrate (0.0328 mmol and the fatty acid in excess (100 molar eq.) were added to the solution. The reaction was initiated by addition of *Ca*lB immobilized on Immobead 150, recombinant from yeast 2000 PLU g^−1^ (147.7 mg). The reaction mixture was heated at 50 °C for 72 h without mechanical stirring. The solution was then allowed to cool and filtered through cotton wool. What remained on the filter was then washed with acidified methanol (0.1% HCl; *ca*. 30 mL) and the methanolic solution was extracted with hexane (3 × 30 mL) to eliminate the excess of fatty acid. The methanolic phase was evaporated under reduced pressure and its residue was analysed with reversed-phase liquid chromatography coupled with mass spectrometer (RP-LC-MS), allowing calculation of the % conversion of each reaction from the obtained chromatograms by integrating the peak area of the acylated and non-acylated parent compound. The residue of the methanolic phase was then dissolved in acidified water (pH ≈ 1.8 with HCl; 15 mL) and extracted with EtOAc (3 × 30 mL). The final organic phase (ethyl acetate) was also analysed with (RP-LC-MS), after evaporation of the solvent under reduced pressure to verify that the majority of the remaining unreacted substrate is eliminated by the second phase of the workup. The results along with some conversion ratios calculated for these first reactions are summarized in entries 1–6 of Table 1. In some of them no product was detected, hence no conversion of the substrate occurred (stated as “Nil”) whereas in some others the acylated product was formed, with the calculated conversion ratios indicating the amount of it. In those cases, some by-products were detected as well in the chromatograms.

### 4.10. Optimisation of the reaction conditions

For optimization of the reaction, numerous trials were performed by modifying and investigating parameters such as the fatty acid (nature and quantity), the solvent system, the dehydrating agent, temperature, and duration. Data such as reaction results, observations and conversion ratios for this procedure are summarized in Tables 1–4.

### 4.11. Optimised protocol for esterification of rutin

Silicon dioxide (40–60 μm; 1.215 g) was added to a solvent mixture of 10 : 1 acetonitrile : DMSO (36.95 mL) and stirred for 30 min to remove residual moisture. The flavonoid disaccharide rutin (100 mg; 0.164 mmol) and the fatty acid in excess (100 molar eq.) were added to the solution. The reaction was initiated by the addition of the corresponding amount of *Ca*lB immobilized on acrylic resin, recombinant from *Aspergillus niger* 5000 PLU g^−1^, (295 mg). The reaction mixture was heated at 55 °C for 72 h without any mechanical stirring. The liquor was then filtered through a sintered glass filter, the residue was washed with acidified methanol (0.1% HCl; *ca.* 90 mL) and the combined filtrate was extracted with hexane (3 × 90 mL). The remaining residue of the methanolic phase, after evaporation of methanol, was taken up with acidified water (pH ≈ 1.8 with HCl; 60 mL) and extracted with EtOAc (3 × 90 mL). The residues of the methanolic and the final organic phase (ethyl acetate) were both analysed with RP-LC-MS, as previously stated and the reactions' conversion ratios were calculated by the chromatograms of the methanolic residue, as already described in Section 4.9. The residue of the final organic phase was then submitted to a Reversed Phase Medium Pressure Liquid Chromatography (RP-MPLC) analysis using Biotage system coupled with a UV-detector in order to purify the synthesized compounds; details of the isolation procedure are already given above (Section 4.6). The most important data for the isolated rutin esters are summarized in Tables 6 and 7 whereas more details are given at the end of the experimental section.

### 4.12. Improved preparative method

Silicon dioxide (40–60 μm; 243 mg) was added to a solvent mixture of 10 : 1 acetonitrile : DMSO (7.39 mL) and stirred for 30 min to remove residual moisture. Rutin (60 mg; 0.0984 mmol) and the fatty acid in excess (10 molar eq.) were added to the solution. The reaction was initiated by addition of the corresponding amount of *Ca*lB immobilized on acrylic resin, recombinant from *Aspergillus niger* 5000 PLU g^−1^, (59 mg). The reaction mixture was heated at 55 °C for 72 h without any mechanical stirring. The liquor was then filtered through a sintered glass filter, the residue was washed with acidified methanol (0.1% HCl; *ca.* 50 mL) and the combined filtrate was extracted with hexane (3 × 30 mL). The remaining residue of the methanolic phase, after evaporation of methanol, was taken up with acidified water (pH ≈ 1.8 with HCl; 30 mL) and extracted with EtOAc (3 × 50 mL). The residues of the methanolic and the final organic phase (ethyl acetate) were both analysed with RP-LC-MS and the reactions' conversion ratios were calculated by the chromatograms of the methanolic residue, as already described in Section 4.9. The reactions' conversion ratios are stated in Table 8. In all cases, no by-products were detected in the crude reaction mixture.

### 4.13. Isolated rutin esters

Herein are listed the analytical data for the synthesised compounds. Copies of the mass and UV-vis spectra are presented in the ESI† along with characteristic areas of the NMR spectra for each of the compounds. Relevant spectroscopic data for the parent compound, rutin, are included as well.

#### 4.13.1 Rutin butyrate (3a)

Synthesized as described in Section 4.11, by esterifying rutin with 100 molar eq. of butyric acid. The enzymatic synthesis of the compound has been reported previously by Viskupicova *et al.*,^31^ however, no spectroscopic data were given.

Yellow solid; C_31_H_38_O_17_; mass: 680.6073 (calc.); 681.47 [M + H]^+^ (found); yield: 23%;

^1^H-NMR (acetone-d_6_, 400 MHz): *δ* 12.47 (bs, H-5), 7.80 (d, H-2′, *J* = 2.1 Hz), 7.71 (dd, H = 6′, *J* = 8.5, 2.1 Hz), 6.97 (d, H-5′, *J* = 8.5 Hz), 6.50 (d, H-8, *J* = 2.0 Hz), 6.28 (d, H-6, *J* = 2.0 Hz), 5.36 (d, H-1′′, *J* = 7.2 Hz), 4.81 (t, H-4′′′, *J* = 9.2 Hz), 4.62 (s, H-1′′′), 3.87 (dd, H-6′′α, *J* = 10.9, 1.0 Hz), 3.70 (bs, H-2′′′), 3.69 (m, H-3′′′), 3.62 (m, H-5′′′), 3.52 (m, H-6′′β), 3.48 (m, H-2′′, H-3′′), 3.43 (m, H-5′′), 3.39 (m, H-4′′), 2.24 (t, CH_2_α, *J* = 7.4 Hz), 1.57 (sext, CH_2_β, *J* = 7.4 Hz), 0.91 (m, CH_3(rha)_), 0.90 (t, CH_3(FA)_, *J* = 6.9 Hz).

^13^C-NMR (MeOD, 100 MHz): *δ* 122.47 (C6′), 117.61 (C2′), 115.83 (C5′), 103.02 (C1′′), 101.40 (C1′′′), 98.85 (C6), 94.41 (C8), 77.39 (C3′′), 76.00 (C5′′), 75.78 (C2′′), 74.26 (C4′′′), 70.24 (C4′′), 73.10 (C3′′′), 72.64 (C2′′′), 68.80 (C6′′), 66.86 (C5′′′), 33.33 (CH_2_α), 25.82 (CH_2β_), 17.88 (CH_3(rha)_), 22.65 (CH_3(FA)_).

UV/vis (1 : 1 v/v methanol : water): *λ*_max_ 291, 309, 343 nm.

#### 4.13.2 Rutin octanoate (3b)

Synthesized as described in Section 4.11, by esterifying rutin with 100 molar eq. of octanoic acid. The enzymatic synthesis of the compound has been already reported by Viskupicova *et al.*,^31^ but no spectroscopic data are available.

Yellow solid; C_35_H_44_O_17_; mass: 736.7137 (calc.); 737.53 [M + H]^+^ (found); yield: 47%;

^1^H-NMR (acetone-d_6_, 400 MHz): *δ* 12.44 (bs, H-5), 7.79 (d, H-2′, *J* = 2.2 Hz), 7.69 (dd, H = 6′, *J* = 8.5, 2.2 Hz), 6.97 (d, H-5′, *J* = 8.5 Hz), 6.51 (d, H-8, *J* = 2.1 Hz), 6.27 (d, H-6, *J* = 2.1 Hz), 5.33 (d, H-1′′, *J* = 7.2 Hz), 4.81 (t, H-4′′′, *J* = 9.5 Hz), 4.61 (s, H-1′′′), 3.85 (dd, H-6′′α, *J* = 10.8, 1.3 Hz), 3.69 (bs, H-2′′′), 3.68 (dd, H-3′′′, *J* = 9.5, 3.4 Hz), 3.61 (dq, H-5′′′, *J* = 9.5, 6.3 Hz), 3.51 (dd, H-6′′β, *J* = 10.8, 5.7 Hz), 3.48 (m, H-2′′, H-3′′), 3.43 (dd, H-5′′, *J* = 5.7, 1.3 Hz), 3.38 (t, H-4′′, *J* = 9.0 Hz), 2.27 (td, CH_2_α, *J* = 7.4, 3.2 Hz), 1.56 (m, CH_2β_), 1.29 (bs, CH_2(FA)_), 0.92 (d, CH_3(rha)_, *J* = 6.3 Hz), 0.87 (t, CH_3(FA),_*J* = 6.9 Hz).

^13^C-NMR (acetone-d_6_, 100 MHz): *δ* 175.11 (C4), 174.96 (–C=O), 165.16 (C7), 163.61 (C5), 158.69 (C2), 158.29 (C9), 149.69 (C4′), 145.69 (C3′), 135.85 (C3), 123.03 (C1′), 122.78 (C6′), 117.32 (C2′), 114.97 (C5′), 105.71 (C10), 103.81 (C1′′), 101.28 (C1′′′), 99.32 (C6), 94.25 (C8), 77.63 (C3′′), 76.77 (C5′′), 75.35 (C2′′), 74.61 (C4′′′), 70.44 (C4′′), 70.22 (C2′′′, C3′′′), 68.20 (C6′′), 66.63 (C5′′′), 34.41 (CH_2_α), 29.31 (CH_2(FA)_), 25.20 (CH_2β_), 23.85 (CH_2(FA)_), 17.33 (CH_3(rha)_), 14.64 (CH_3(FA)_).

UV/vis (1 : 1 v/v methanol : water): *λ*_max_ 291, 348, 355 nm.

#### 4.13.3 Rutin laureate (3c)

Synthesized as described in Section 4.11, by esterifying rutin with 100 molar eq. of dodecanoic acid. The synthesis of the compound has been reported enzymatically by Viskupicova *et al.*^31^ and Lue *et al.*,^2^ both providing some spectroscopic data. The obtained data during this work agree to some extent with the literature values.

Yellow solid; C_39_H_52_O_17_; mass: 792.8200 (calc.); 793.25 [M + H]^+^ (found); yield: 41%;

^1^H-NMR (acetone-d_6_, 400 MHz): *δ* 12.45 (bs, H-5), 7.78 (d, H-2′, *J* = 2.1 Hz), 7.70 (dd, H = 6′, *J* = 8.5, 2.1 Hz), 6.97 (d, H-5′, *J* = 8.5 Hz), 6.50 (d, H-8, *J* = 2.1 Hz), 6.27 (d, H-6, *J* = 2.1 Hz), 5.34 (d, H-1′′, *J* = 7.3 Hz), 4.81 (t, H-4′′′, *J* = 9.5 Hz), 4.61 (s, H-1′′′), 3.85 (dd, H-6′′α, *J* = 10.7, 1.1 Hz), 3.70 (bs, H-2′′′), 3.68 (dd, H-3′′′, *J* = 9.5, 3.3 Hz), 3.60 (dq, H-5′′′, *J* = 9.5, 6.3 Hz), 3.51 (dd, H-6′′β, *J* = 10.7, 5.7 Hz), 3.47 (m, H-2′′, H-3′′), 3.42 (dd, H-5′′, *J* = 5.7, 1.1 Hz), 3.38 (m, H-4′′), 2.27 (td, CH_2_α, *J* = 7.3, 3.2 Hz), 1.55 (m, CH_2β_), 1.28 (bs, CH_2(FA)_), 0.92 (d, CH_3(rha)_, *J* = 6.3 Hz), 0.86 (t, CH_3(FA),_*J* = 6.8 Hz).

^13^C-NMR (acetone-d_6_, 100 MHz): *δ* 175.95 (C4), 174.46 (–C=O), 165.52 (C7), 163.32 (C5), 159.36 (C2), 158.09 (C9), 150.14 (C4′), 145.52 (C3′), 131.75 (C3), 123.86 (C1′), 122.94 (C6′), 117.32 (C2′), 115.74 (C5′), 105.58 (C10), 104.04 (C1′′), 101.46 (C1′′′), 99.42 (C6), 94.43 (C8), 77.93 (C3′′), 76.57 (C5′′), 75.15 (C2′′), 74.49 (C4′′′), 71.61 (C2′′′, C3′′′), 69.64 (C4′′), 67.92 (C6′′), 66.63 (C5′′′), 34.46 (CH_2_α), 30.11 (CH_2(FA)_), 25.46 (CH_2β_), 23.30 (CH_2(FA)_), 17.54 (CH_3(rha)_), 14.11 (CH_3(FA)_).

UV/vis (1 : 1 v/v methanol : water): *λ*_max_ 263, 291, 354 nm.

#### 4.13.4 Rutin palmitate (3d)

Synthesized as described in Section 4.11, by esterifying rutin with 100 molar eq. of hexadecanoic acid. Several syntheses have been accomplished previously,^2,31,32^ and in all cases 3d was obtained enzymatically. Spectroscopic data are provided in some cases.

Yellow solid; C_43_H_60_O_17_; mass: 848.9263 (calc.); 849.49 [M + H]^+^ (found); yield: 33%;

^1^H-NMR (acetone-d_6_, 400 MHz): *δ* 12.47 (bs, H-5), 7.78 (d, H-2′, *J* = 2.1 Hz), 7.70 (dd, H = 6′, *J* = 8.5, 2.1 Hz), 6.97 (d, H-5′, *J* = 8.5 Hz), 6.50 (d, H-8, *J* = 2.1 Hz), 6.27 (d, H-6, *J* = 2.1 Hz), 5.34 (d, H-1′′, *J* = 7.3 Hz), 4.81 (t, H-4′′′, *J* = 9.5 Hz), 4.61 (s, H-1′′′), 3.85 (dd, H-6′′α, *J* = 10.9, 1.2 Hz), 3.70 (bs, H-2′′′), 3.68 (dd, H-3′′′, *J* = 9.5, 3.2 Hz), 3.60 (dq, H-5′′′, *J* = 9.5, 6.3 Hz), 3.51 (dd, H-6′′β, *J* = 10.9, 5.6 Hz), 3.47 (m, H-2′′, H-3′′), 3.42 (dd, H-5′′, *J* = 5.7, 1.2 Hz), 3.38 (m, H-4′′), 2.27 (td, CH_2_α, *J* = 7.5, 3.2 Hz), 1.56 (m, CH_2β_), 1.28 (bs, CH_2(FA)_), 0.92 (d, CH_3(rha)_, *J* = 6.3 Hz), 0.87 (t, CH_3(FA),_*J* = 6.7 Hz).

^13^C-NMR (acetone-d_6_, 100 MHz): *δ* 176.60 (C4), 174.19 (–C=O), 165.40 (C7), 162.78 (C5), 158.74 (C2), 158.02 (C9), 149.30 (C4′), 145.06 (C3′), 131.19 (C3), 123.18 (C1′), 122.74 (C6′), 117.32 (C2′), 115.56 (C5′), 105.14 (C10), 103.90 (C1′′), 101.43 (C1′′′), 99.55 (C6), 94.47 (C8), 77.87 (C3′′), 76.61 (C5′′), 75.40 (C2′′), 75.08 (C4′′′), 71.23 (C2′′′, C3′′′), 70.42 (C4′′), 67.68 (C6′′), 66.27 (C5′′′), 34.13 (CH_2_α), 30.00 (CH_2(FA)_), 25.73 (CH_2(FA)_), 25.33 (CH_2β_), 23.31 (CH_2(FA)_), 17.45 (CH_3(rha)_), 14.07 (CH_3(FA)_).

UV/vis (1 : 1 v/v methanol : water): *λ*_max_ 291, 303, 368, 409 nm.

#### 4.13.5 Rutin stearate (3e)

Synthesized as described in Section 4.11, by esterifying rutin with 100 molar eq. of octadecanoic acid. Several enzymatic syntheses of 3e have been accomplished previously,^21,31,32^ with the first one^21^ to provide both ^1^H and ^13^C NMR data for the synthesized analogue.

Yellow solid; C_45_H_64_O_17_; mass: 876.9795 (calc.); 877.40 [M + H]^+^ (found); yield: 10%;

^1^H-NMR (acetone-d_6_, 400 MHz): *δ* 12.47 (bs, H-5), 7.80 (d, H-2′, *J* = 2.1 Hz), 7.69 (dd, H = 6′, *J* = 8.5, 2.1 Hz), 6.98 (d, H-5′, *J* = 8.5 Hz), 6.50 (d, H-8, *J* = 2.0 Hz), 6.28 (d, H-6, *J* = 2.0 Hz), 5.35 (d, H-1′′, *J* = 7.2 Hz), 4.81 (t, H-4′′′, *J* = 9.4 Hz), 4.61 (s, H-1′′′), 3.87 (dd, H-6′′α, *J* = 11.0, 1.2 Hz), 3.69 (bs, H-2′′′), 3.68 (dd, H-3′′′, *J* = 9.4, 3.6 Hz), 3.60 (dq, H-5′′′, *J* = 9.4, 6.2 Hz), 3.51 (dd, H-6′′β, *J* = 11.0, 5.8 Hz), 3.49 (m, H-3′′), 3.47 (m, H-2′′), 3.43 (dd, H-5′′, *J* = 5.8, 1.2 Hz), 3.38 (m, H-4′′), 2.27 (t, CH_2_α, *J* = 7.1 Hz), 1.55 (m, CH_2β_), 1.28 (bs, CH_2(FA)_), 0.92 (d, CH_3(rha)_, *J* = 6.2 Hz), 0.87 (t, CH_3(FA),_*J* = 6.7 Hz).

^13^C-NMR (acetone-d_6_, 100 MHz): *δ* 122.87 (C6′), 117.36 (C2′), 115.35 (C5′), 103.89 (C1′′), 101.42 (C1′′′), 99.46 (C6), 94.40 (C8), 77.82 (C3′′), 76.30 (C5′′), 75.40 (C2′′), 74.66 (C4′′′), 71.52 (C2′′′, C3′′′), 70.59 (C4′′), 67.59 (C6′′), 66.49 (C5′′′), 34.21 (CH_2_α), 30.14 (CH_2(FA)_), 25.46 (CH_2β_), 22.45 (CH_2(FA)_), 17.48 (CH_3(rha)_), 13.70 (CH_3(FA)_).

UV/vis (1 : 1 v/v methanol : water): *λ*_max_ 291, 308, 363 nm.

#### 4.13.6 Rutin oleate (3f)

Synthesized as described in Section 4.11, by esterifying rutin with 100 molar eq. of oleic (*cis*-9-octadecenoic) acid. The enzymatic synthesis of the compound has been already reported by Zheng *et al.*,^4^ but no spectroscopic data are available.

Yellow solid; C_45_H_62_O_17_; mass: 874.9636 (calc.); 875.18 [M + H]^+^ (found); yield: 27%;

^1^H-NMR (acetone-d_6_, 400 MHz): *δ* 12.47 (bs, H-5), 7.80 (d, H-2′, *J* = 2.1 Hz), 7.70 (dd, H = 6′, *J* = 8.4, 2.1 Hz), 6.98 (d, H-5′, *J* = 8.4 Hz), 6.50 (d, H-8, *J* = 1.9 Hz), 6.28 (d, H-6, *J* = 1.9 Hz), 5.35 (d, H-1′′, *J* = 7.0 Hz), 5.34 (m, –CH=CH–), 4.81 (t, H-4′′′, *J* = 9.5 Hz), 4.61 (s, H-1′′′), 3.87 (dd, H-6′′α, *J* = 10.9, 1.1 Hz), 3.70 (bs, H-2′′′), 3.67 (dd, H-3′′′, *J* = 9.5, 3.3 Hz), 3.61 (dq, H-5′′′, *J* = 9.5, 6.2 Hz), 3.52 (dd, H-6′′β, *J* = 10.9, 5.7 Hz), 3.48 (m, H-3′′), 3.45 (m, H-2′′), 3.43 (dd, H-5′′, *J* = 5.7, 1.1 Hz), 3.38 (t, H-4′′, *J* = 8.7 Hz), 2.27 (t, CH_2_α, *J* = 7.4 Hz), 2.08 (bs, –CH_2α_CH=), 1.59 (quint, CH_2β_, *J* = 7.1 Hz), 1.30 (bs, CH_2(FA)_), 0.92 (d, CH_3(rha)_, *J* = 6.2 Hz), 0.88 (t, CH_3(FA),_*J* = 6.8 Hz).

^13^C-NMR (acetone-d_6_, 100 MHz): *δ* 177.66 (C4), 174.72 (–C=O), 164.95 (C7), 162.94 (C5), 158.50 (C2), 157.41 (C9), 150.58 (C4′), 145.50 (C3′), 135.43 (C3), 130.30 (–CH=), 123.55 (C1′), 123.01 (C6′), 117.20 (C2′), 115.58 (C5′), 105.72 (C10), 103.80 (C1′′), 101.42 (C1′′′), 99.39 (C6), 94.39 (C8), 77.90 (C3′′), 76.62 (C5′′), 75.17 (C2′′), 74.45 (C4′′′), 71.63 (C2′′′, C3′′′), 69.70 (C4′′), 67.95 (C6′′), 66.65 (C5′′′), 33.90 (CH_2_α), 29.55 (CH_2(FA)_), 27.54 (–CH_2α_CH=), 25.41 (CH_2β_), 22.82 (CH_2(FA)_), 17.51 (CH_3(rha)_), 14.10 (CH_3(FA)_).

UV/vis (1 : 1 v/v methanol : water): *λ*_max_ 291, 358 nm.

## Conflicts of interest

There are no conflicts to declare.

## Supplementary Material

RA-013-D3RA06333J-s001
