# Supplementary material for: Chemo- and regio-selective enzymatic lipophilisation of rutin, and physicochemical and antioxidant properties of rutin ester derivatives
Source: RSC Adv. 2023 Dec 4;13(50):35216–30. doi: 10.1039/d3ra06333j (PMC10694792; doi:10.1039/d3ra06333j)
Supplement: RA-013-D3RA06333J-s001 [file RA-013-D3RA06333J-s001.pdf]

Electronic Supplementary Material (ESI) for RSC Advances.
This journal is © The Royal Society of Chemistry 2023

Chemo- and Regio-selective Enzymatic Lipophilisation of Rutin, and Physicochemical and Antioxidant Properties of Rutin Ester Derivatives

Nikitia Mexia,[a,b] Meryem Benohoud,[c] Christopher M. Rayner,[b,c] Richard S. Blackburn*[a,c]

[a]Leeds Institute of Textiles and Colour, School of Design, University of Leeds, Leeds, LS2 9JT, UK. E-mail: r.s.blackburn@leeds.ac.uk; [b]School of Chemistry, University of Leeds, Leeds, LS2 9JT, UK; [c]Keracol Limited, Nexus, Discovery Way, Leeds, LS2 3AA, UK.

Supporting Information

NMR Spectra

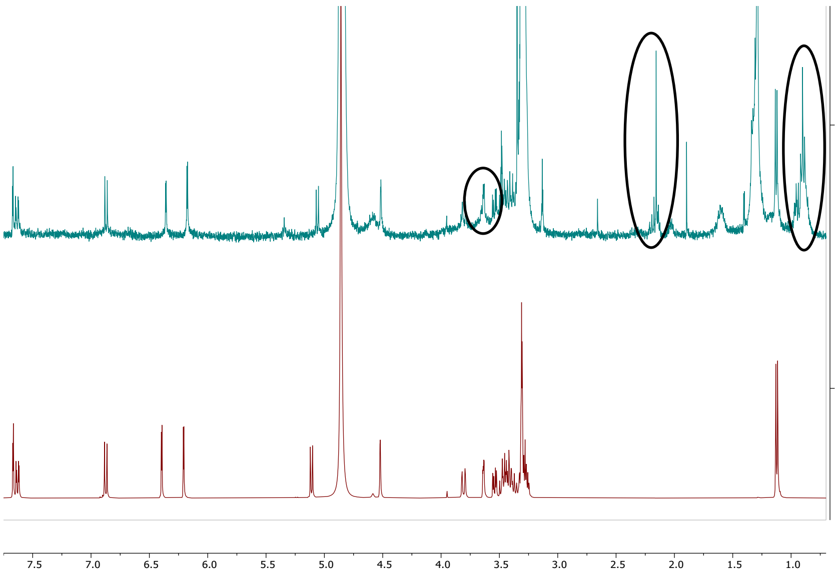

Figure S.1. ^{1}H-NMR of rutin (**2**; Red) and rutin butyrate (**3a**; Green) in MeOD with some significant differences marked.

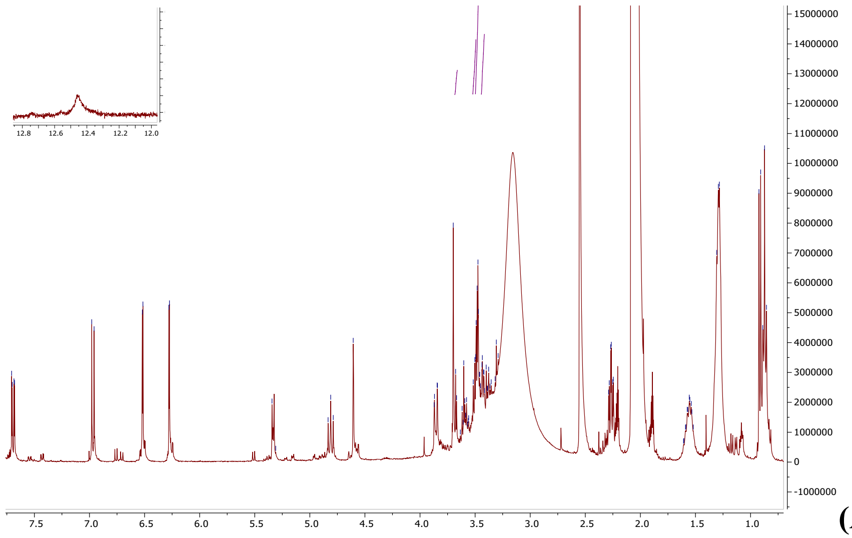
12.8 12.6 12.4 12.2 12.0
15000000 14000000 13000000 12000000 11000000 10000000 9000000 8000000 7000000 6000000 5000000 4000000 3000000 2000000 1000000 0 -1000000
7.5 7.0 6.5 6.0 5.5 5.0 4.5 4.0 3.5 3.0 2.5 2.0 1.5 1.0

(A)

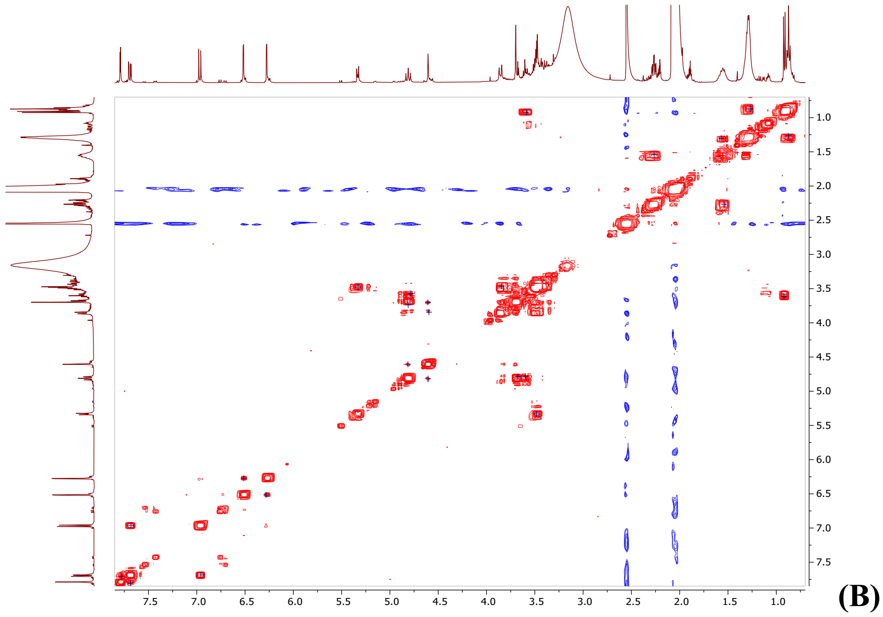
1.0 1.5 2.0 2.5 3.0 3.5 4.0 4.5 5.0 5.5 6.0 6.5 7.0 7.5
7.5 7.0 6.5 6.0 5.5 5.0 4.5 4.0 3.5 3.0 2.5 2.0 1.5 1.0

(B)

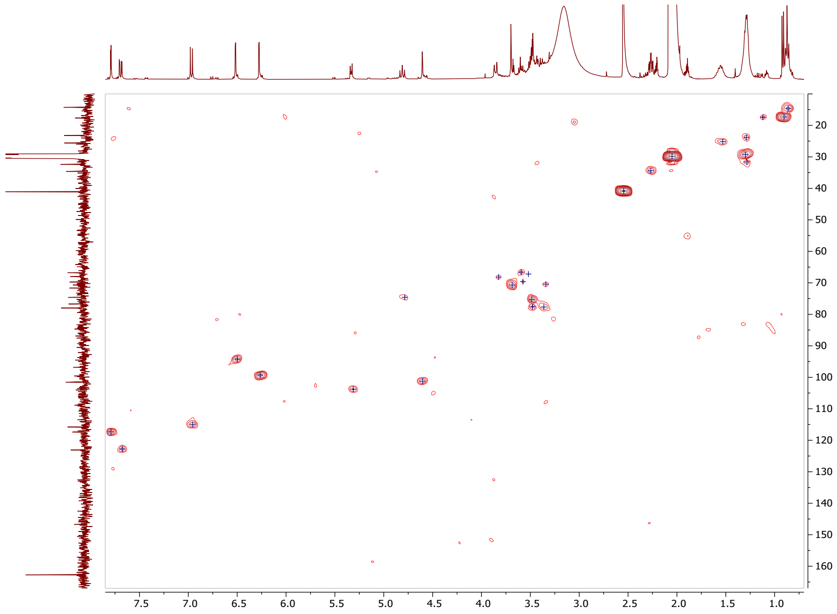
20 30 40 50 60 70 80 90 100 110 120 130 140 150 160
7.5 7.0 6.5 6.0 5.5 5.0 4.5 4.0 3.5 3.0 2.5 2.0 1.5 1.0

(C)

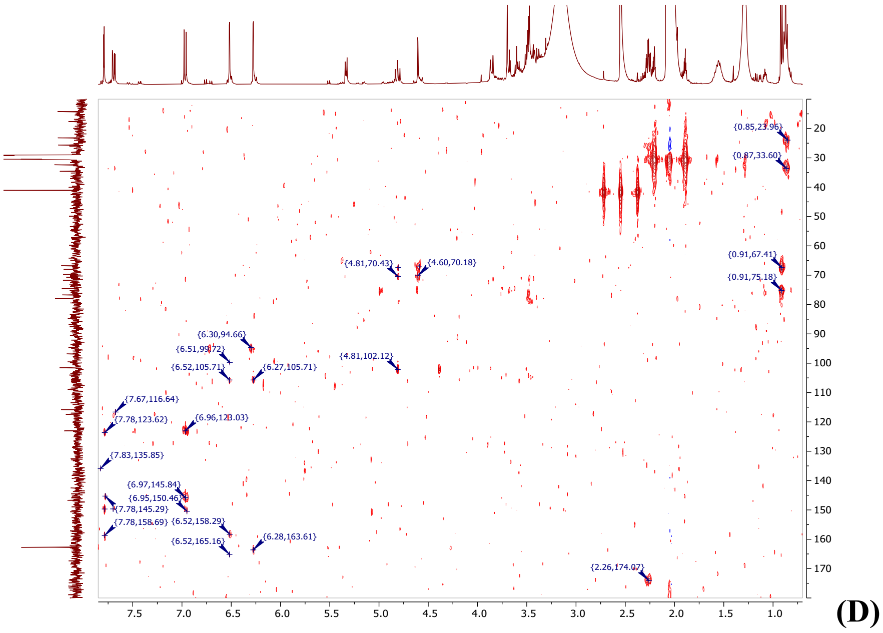

(D)

Figure S.2. Full NMR spectra of rutin octanoate (**3b**) in acetone-d_6 **(A)** ^{1}H-NMR with expansion for the 12-12.8ppm area; **(B)** COSY; **(C)** HMQC; **(D)** HMBC.

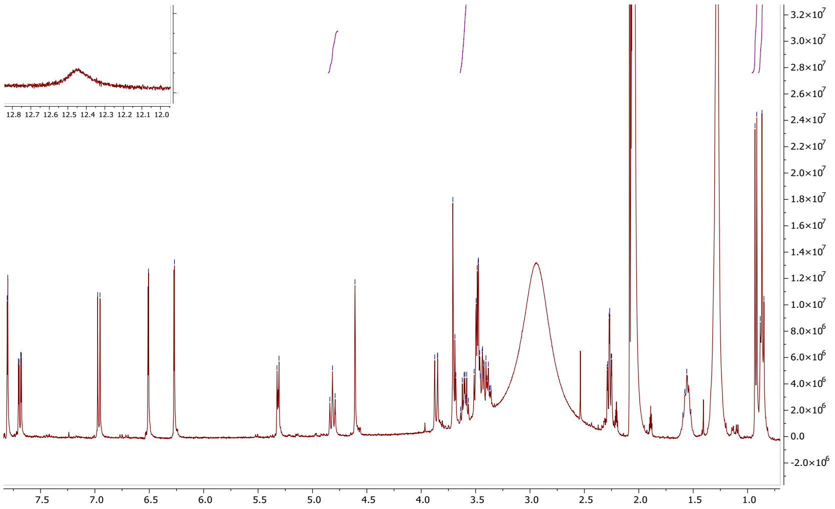

(A)

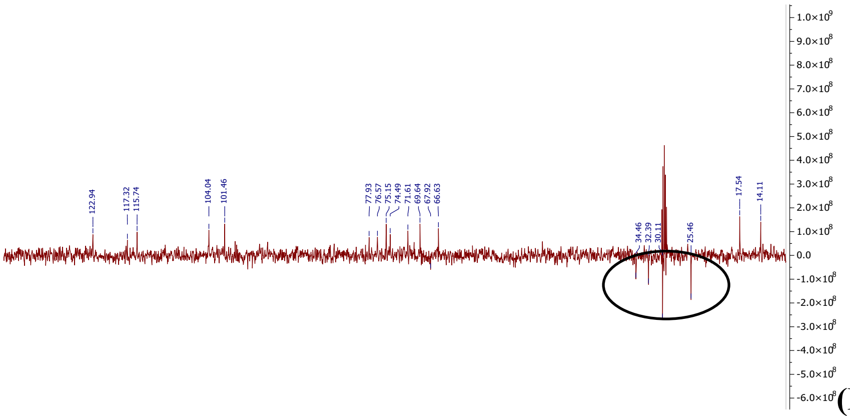

(B)

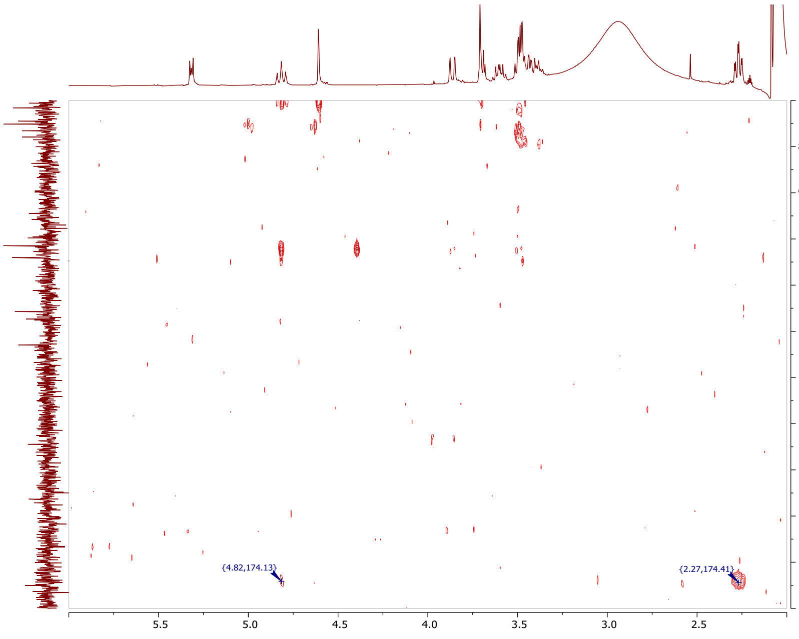

(C)

Figure S.3. Indicative NMR spectra of rutin laureate (**3c**) in acetone-d_6 **(A)** ^{1}H-NMR with expansion for the 12-12.8ppm area; **(B)** ^{13}C-DEPT (marked are the -CH_2- groups); **(C)** HMBC (marked are the signals relating H-4''' and -CH_2aC=O with the same carbonyl group, indicating the coupling between rutin and the fatty acid chain).

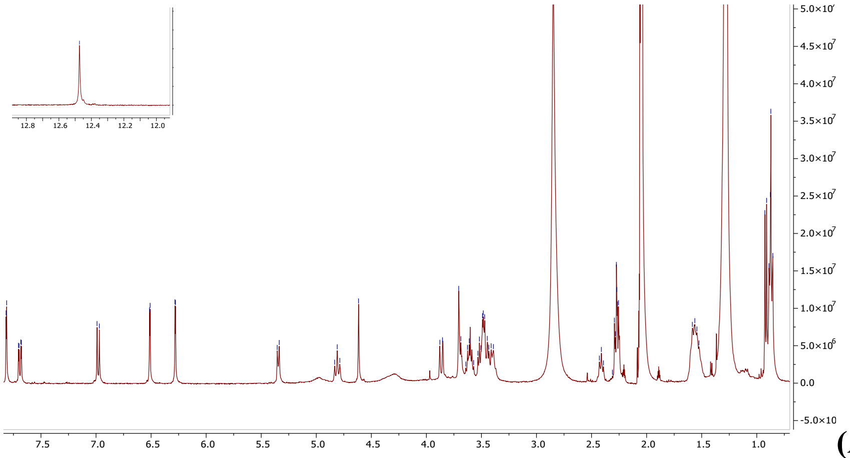

(A)

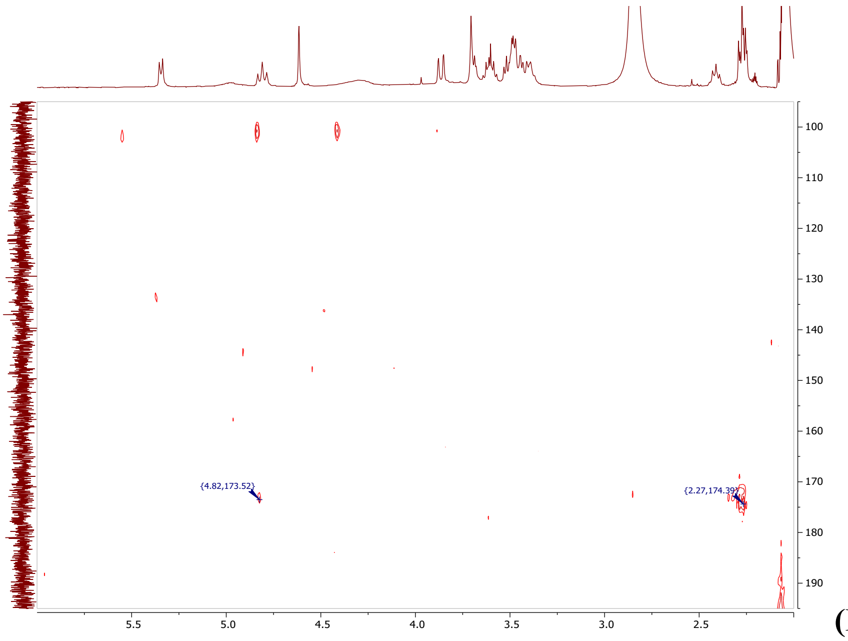

(B)

Figure S.4. Indicative NMR spectra of rutin palmitate (**3d**) in acetone-d_6 **(A)** ^{1}H-NMR with expansion for the 12-12.8ppm area; **(B)** HMBC (marked are the signals relating H-4''' and -CH_2aC=O with the same carbonyl group, indicating the coupling between rutin and the fatty acid chain).

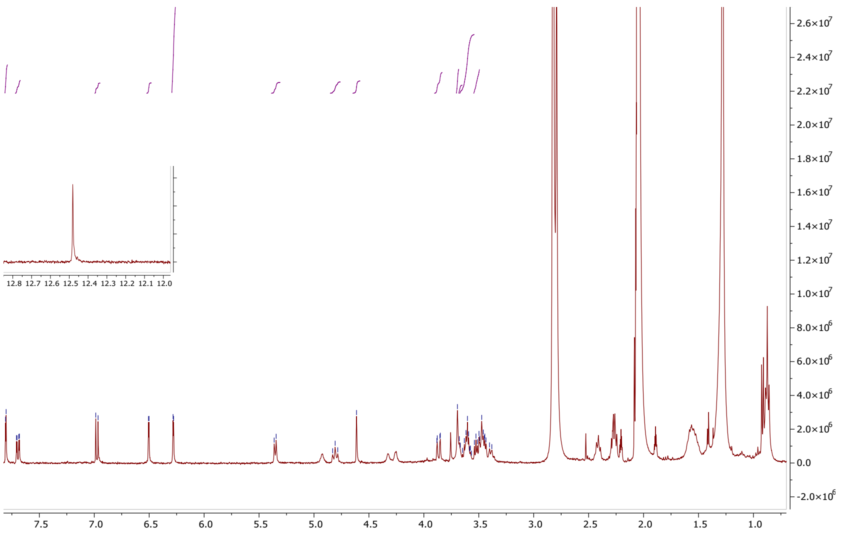

Figure S.5. ^{1}H-NMR spectra of rutin stearate (**3e**) in acetone-d_6 with expansion for the 12-12.8ppm area.

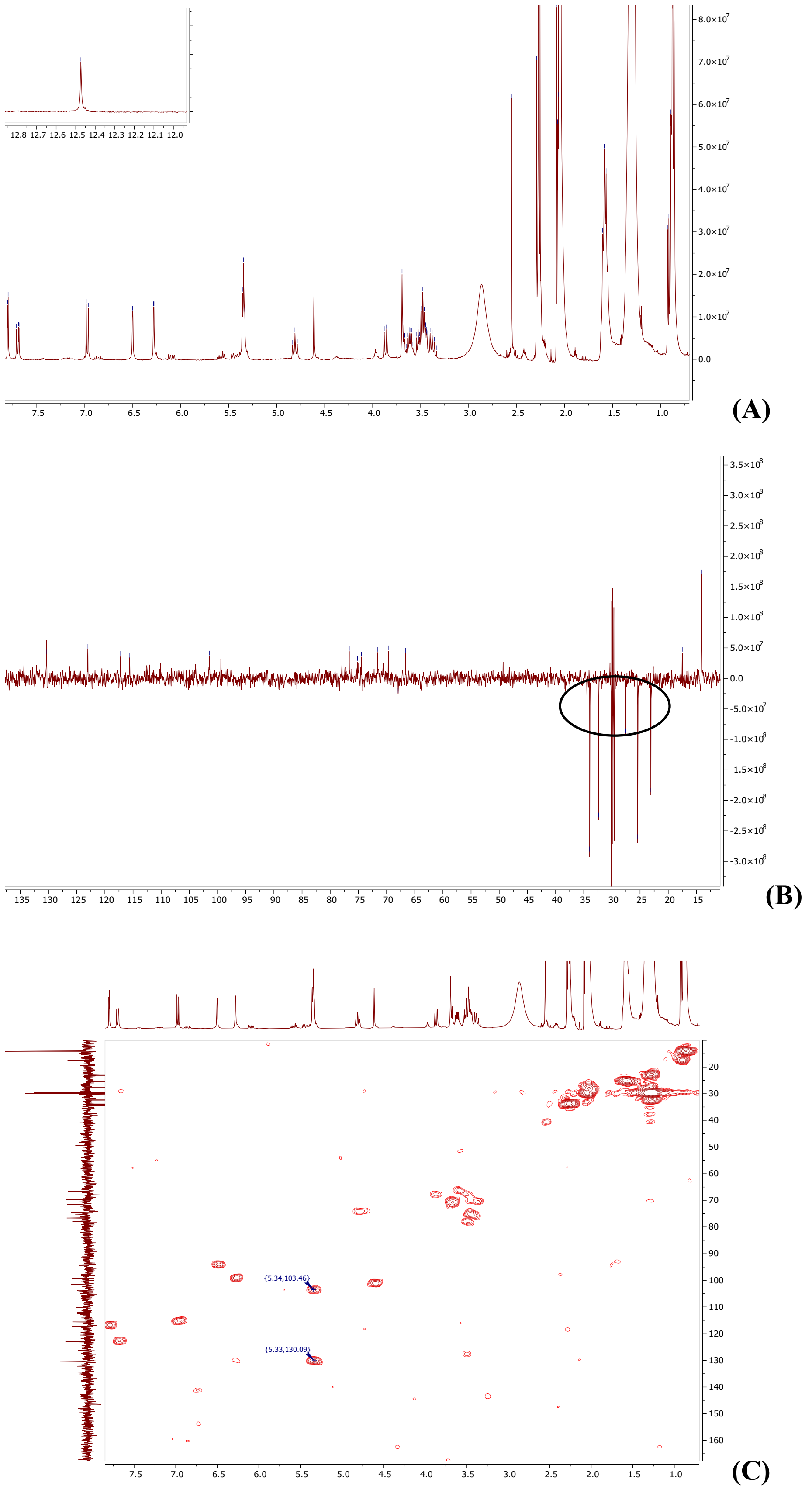

Figure S.6. Indicative NMR spectra of rutin oleate (**3f**) in acetone-d$_6$ **(A)** ^{1}H-NMR with expansion for the 12-12.8ppm area; **(B)** ^{13}C-DEPT (marked are the -CH$_2$- groups); **(C)** HMQC-DEPT (marked are the carbons corresponding to the –CH- groups forming the double bond).

Mass Spectra

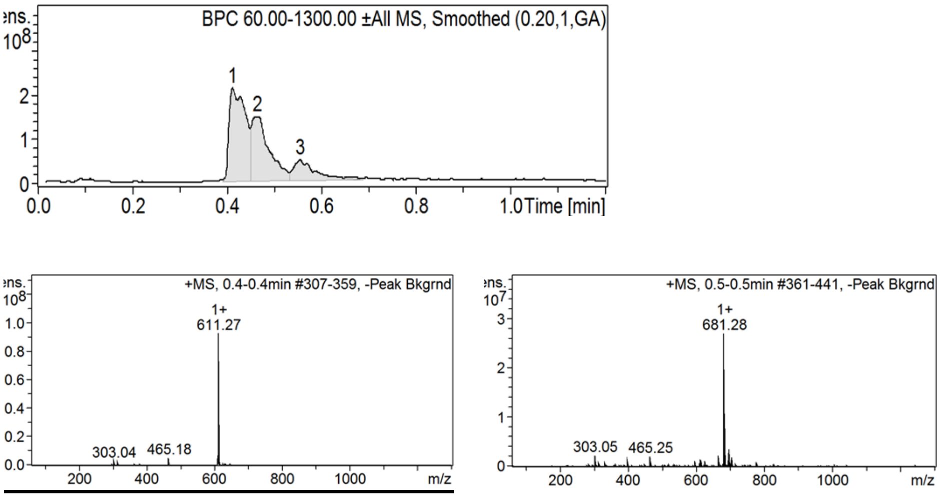

Figure S.7. Mass spectra in methanol of rutin (**2**; peak 1) and rutin butyrate (**3a**; peaks 2-3).

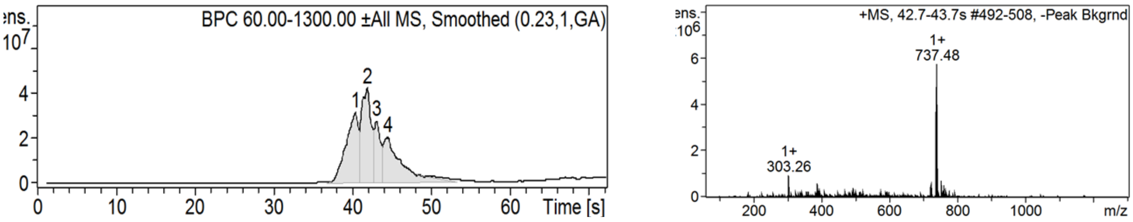

Figure S.8. Mass spectra in methanol of rutin octanoate (**3b**).

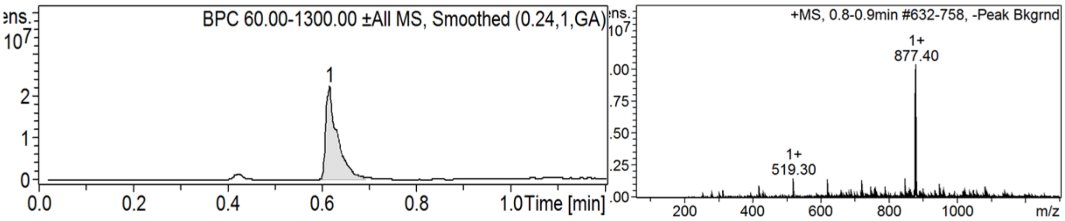

Figure S.9. Mass spectra in methanol of rutin laureate (**3c**).

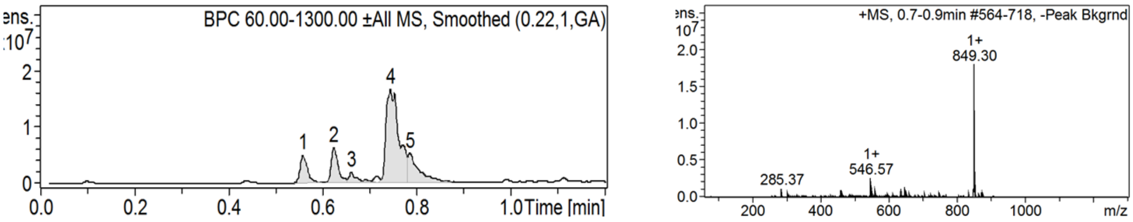

Figure S.10. Mass spectra in methanol of rutin palmitate (**3d** ;peaks 4&5).

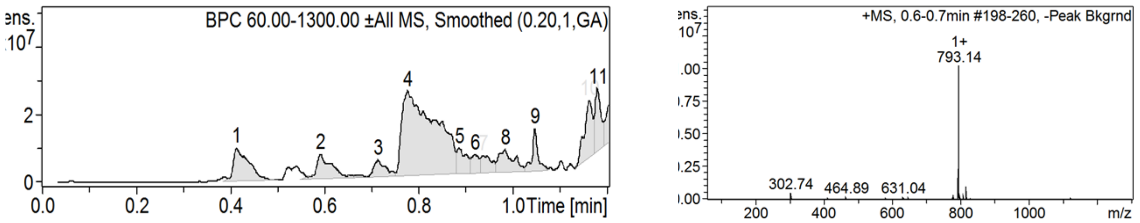

Figure S.11. Mass spectra in methanol of rutin stearate (**3e** ;peaks 3-6).

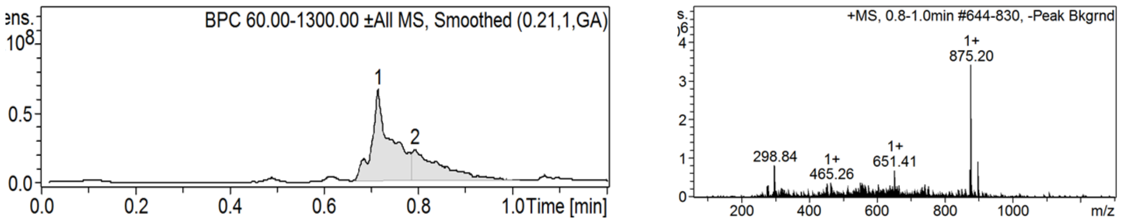

Figure S.12. Mass spectra in methanol of rutin oleate (**3f**).

UV-Vis Spectra

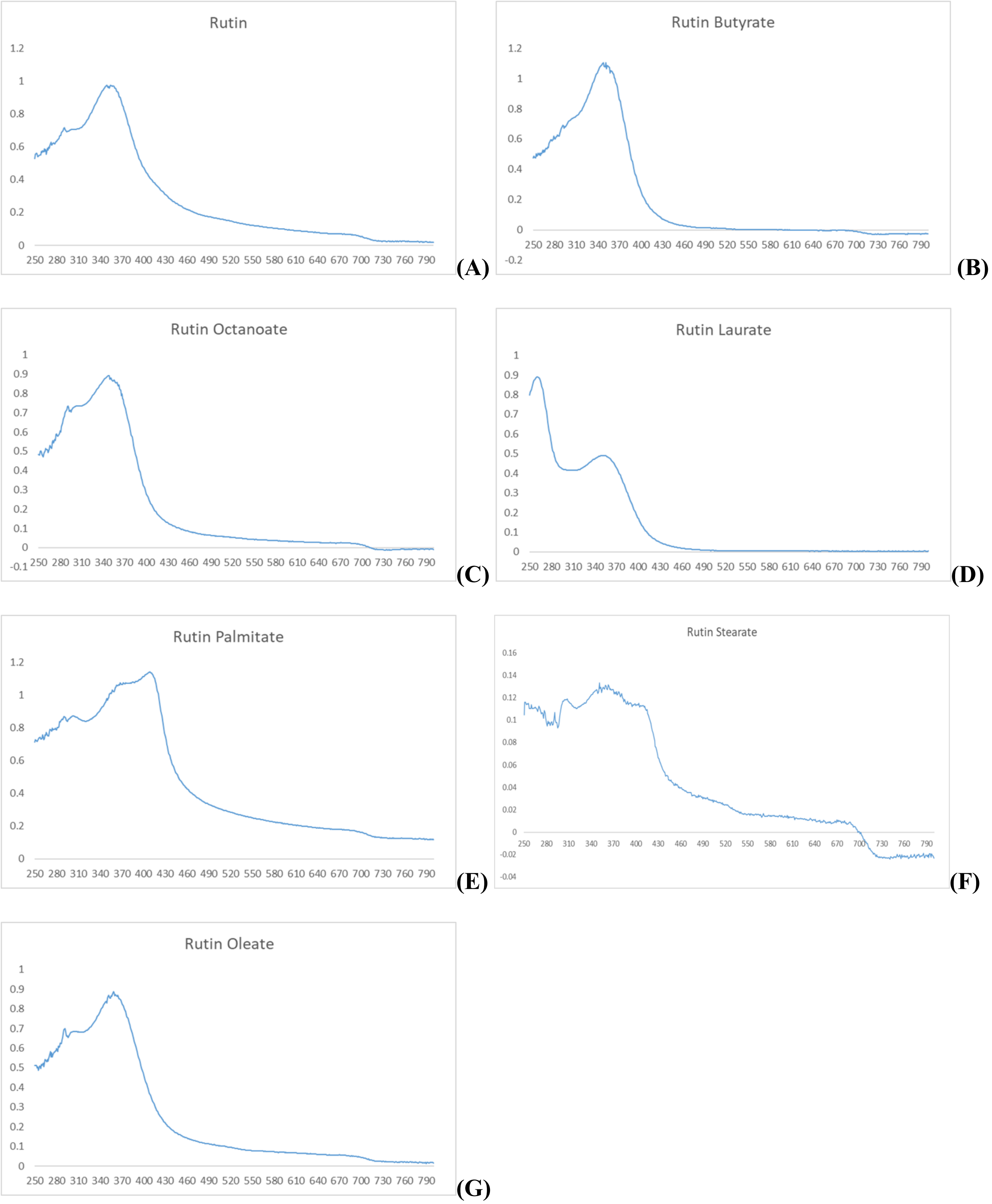

Figure S.13. UV-Vis spectra in 1:1 *v*/*v* methanol:water of: **(A)** rutin (**2**); **(B)** rutin butyrate (**3a**); **(C)** rutin octanoate (**3b**); **(D)** rutin laureate (**3c**); **(E)** rutin palmitate (**3d**); **(F)** rutin stearate (**3e**); **(G)**.rutin oleate (**3f**).

LC-MS Chromatograms for Kinetic Study

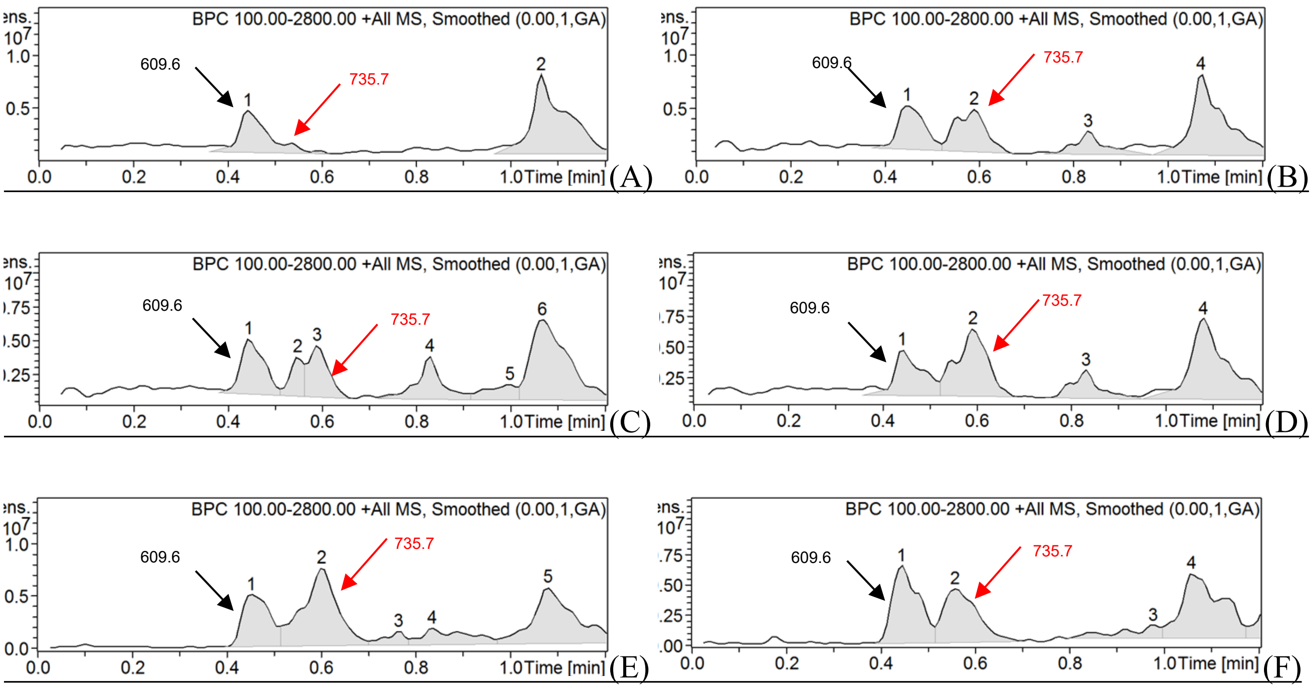

Figure S.14. LC-MS chromatograms for Experiment 45: rutin (4.4 mM) acylation by *Cal*B on acrylic resin with 100 equivalents of octanoic acid in 10:1 acetonitrile:DMSO, at 55 °C with no mechanical stirring after (A) 2 h; (B) 18 h; (C) 24 h; (D) 48 h; (E) 72 h; (F) 96 h. Black arrow indicates peak for rutin (**2**) and red arrow indicates peak for rutin octanoate (**3b**).

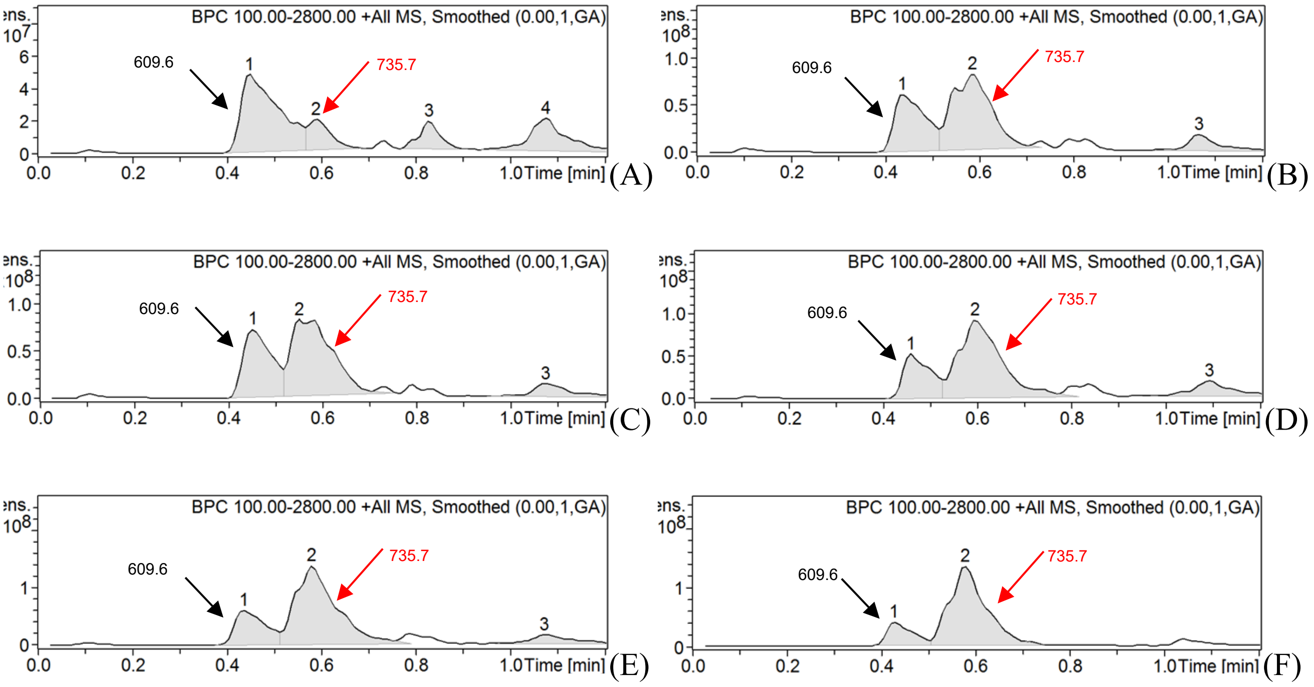

Figure S.15. LC-MS chromatograms for Experiment 46: rutin (13.3 mM) acylation by *Cal*B on acrylic resin with 100 equivalents of octanoic acid in 10:1 acetonitrile:DMSO, at 55 °C with no mechanical stirring after (A) 2 h; (B) 18 h; (C) 24 h; (D) 48 h; (E) 72 h; (F) 96 h. Black arrow indicates peak for rutin (**2**) and red arrow indicates peak for rutin octanoate (**3b**).

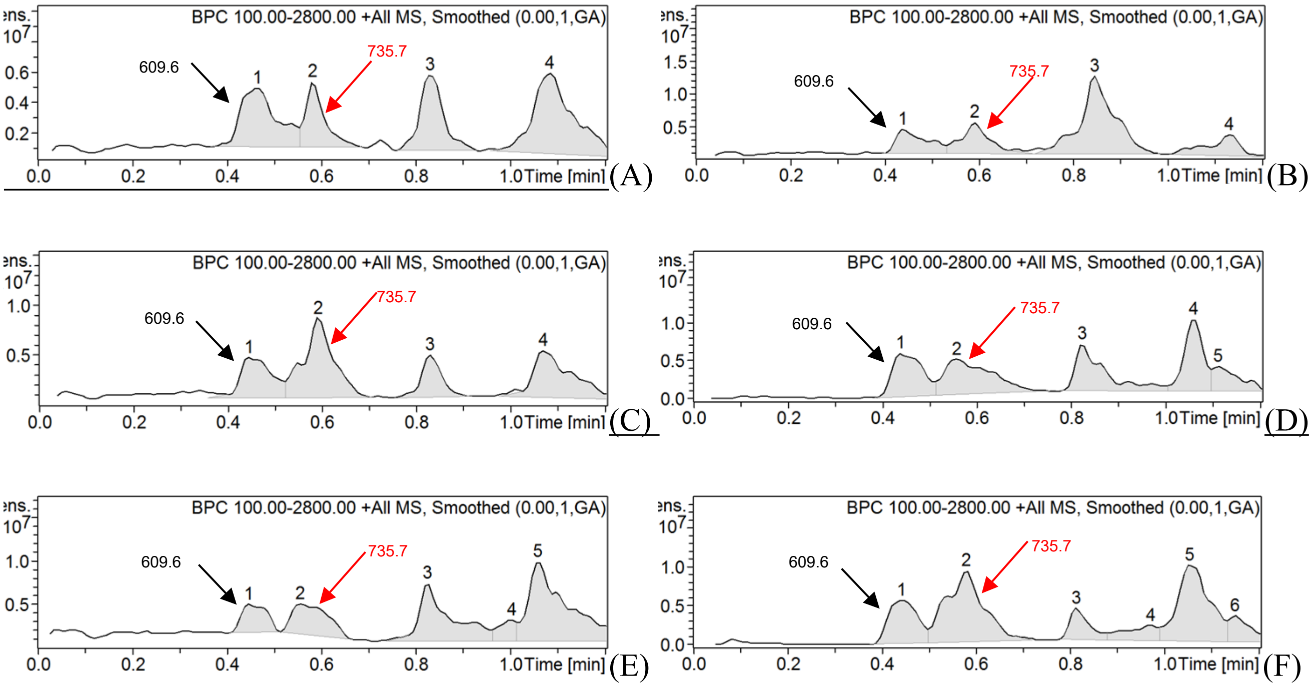

Figure S.16. LC-MS chromatograms for Experiment 47: rutin (22.2 mM) acylation by *Cal*B on acrylic resin with 100 equivalents of octanoic acid in 10:1 acetonitrile:DMSO, at 55 °C with no mechanical stirring after (A) 2 h; (B) 18 h; (C) 24 h; (D) 48 h; (E) 72 h; (F) 96 h. Black arrow indicates peak for rutin (**2**) and red arrow indicates peak for rutin octanoate (**3b**).
